# Comparative assessment of genes driving cancer and somatic evolution in non-cancer tissues: an update of the Network of Cancer Genes (NCG) resource

**DOI:** 10.1186/s13059-022-02607-z

**Published:** 2022-01-26

**Authors:** Lisa Dressler, Michele Bortolomeazzi, Mohamed Reda Keddar, Hrvoje Misetic, Giulia Sartini, Amelia Acha-Sagredo, Lucia Montorsi, Neshika Wijewardhane, Dimitra Repana, Joel Nulsen, Jacki Goldman, Marc Pollitt, Patrick Davis, Amy Strange, Karen Ambrose, Francesca D. Ciccarelli

**Affiliations:** 1 Cancer Systems Biology Laboratory, The Francis Crick Institute, London, NW1 1AT UK; 2 School of Cancer and Pharmaceutical Sciences, King’s College London, London, SE11UL UK; 3 Department of Medical and Molecular Genetics, King’s College London, London, SE1 9RT UK; 4 Scientific Computing, The Francis Crick Institute, London, NW1 1AT UK

**Keywords:** Driver genes, Somatic evolution, Cancer initiation, Systems-level properties

## Abstract

**Background:**

Genetic alterations of somatic cells can drive non-malignant clone formation and promote cancer initiation. However, the link between these processes remains unclear and hampers our understanding of tissue homeostasis and cancer development.

**Results:**

Here, we collect a literature-based repertoire of 3355 well-known or predicted drivers of cancer and non-cancer somatic evolution in 122 cancer types and 12 non-cancer tissues. Mapping the alterations of these genes in 7953 pan-cancer samples reveals that, despite the large size, the known compendium of drivers is still incomplete and biased towards frequently occurring coding mutations. High overlap exists between drivers of cancer and non-cancer somatic evolution, although significant differences emerge in their recurrence. We confirm and expand the unique properties of drivers and identify a core of evolutionarily conserved and essential genes whose germline variation is strongly counter-selected. Somatic alteration in even one of these genes is sufficient to drive clonal expansion but not malignant transformation.

**Conclusions:**

Our study offers a comprehensive overview of our current understanding of the genetic events initiating clone expansion and cancer revealing significant gaps and biases that still need to be addressed. The compendium of cancer and non-cancer somatic drivers, their literature support, and properties are accessible in the Network of Cancer Genes and Healthy Drivers resource at http://www.network-cancer-genes.org/.

**Supplementary Information:**

The online version contains supplementary material available at 10.1186/s13059-022-02607-z.

## Background

Genetic alterations conferring selective advantages to cancer cells are the main drivers of cancer evolution and hunting for them has been at the core of international cancer genomic efforts [1–3]. Given the instability of the cancer genome, distinguishing driver alterations from the rest relies on analytical approaches that identify genes altered more frequently than expected or quantify the positive selection acting on them [4–6]. The results of these analyses have greatly expanded our understanding of the mechanisms driving cancer evolution, revealing high heterogeneity across and within cancers [7–9].

Recently, deep sequencing screens of non-cancer tissues have started to map positively selected genetic mutations in somatic cells that drive in situ formation of phenotypically normal clones [10, 11]. Many of these mutations hit cancer drivers, sometimes at a frequency higher than the corresponding cancer [12–16]. Yet, they do not drive malignant transformation. This conundrum poses fundamental questions on how genetic drivers of normal somatic evolution are related to and differ from those of cancer evolution. Addressing these questions will clarify the genetic relationship between tissue homeostasis and cancer initiation, with profound implications for cancer early detection.

To assess the extent of the current knowledge on cancer and non-cancer drivers, we undertook a systematic review of the literature and assembled a comprehensive repertoire of genes whose somatic alterations have been reported to drive cancer or non-cancer evolution. This allowed us to compare the current driver repertoire across and within cancer and non-cancer tissues and map their alterations in the large pancancer collection of samples from The Cancer Genome Atlas (TCGA). This revealed significant gaps and biases in our current knowledge of the driver landscape. We also computed an array of systems-level properties across driver groups, confirming the unique evolutionary path of driver genes and their central role in the cell.

We collected all cancer and non-cancer driver genes, together with a large set of their properties, in the Network of Cancer Genes and Healthy Drivers (NCG^HD^) open-access resource.

## Results

### More than 3300 genes are canonical or candidate drivers of cancer and non-cancer somatic evolution

**Fig. 1:**
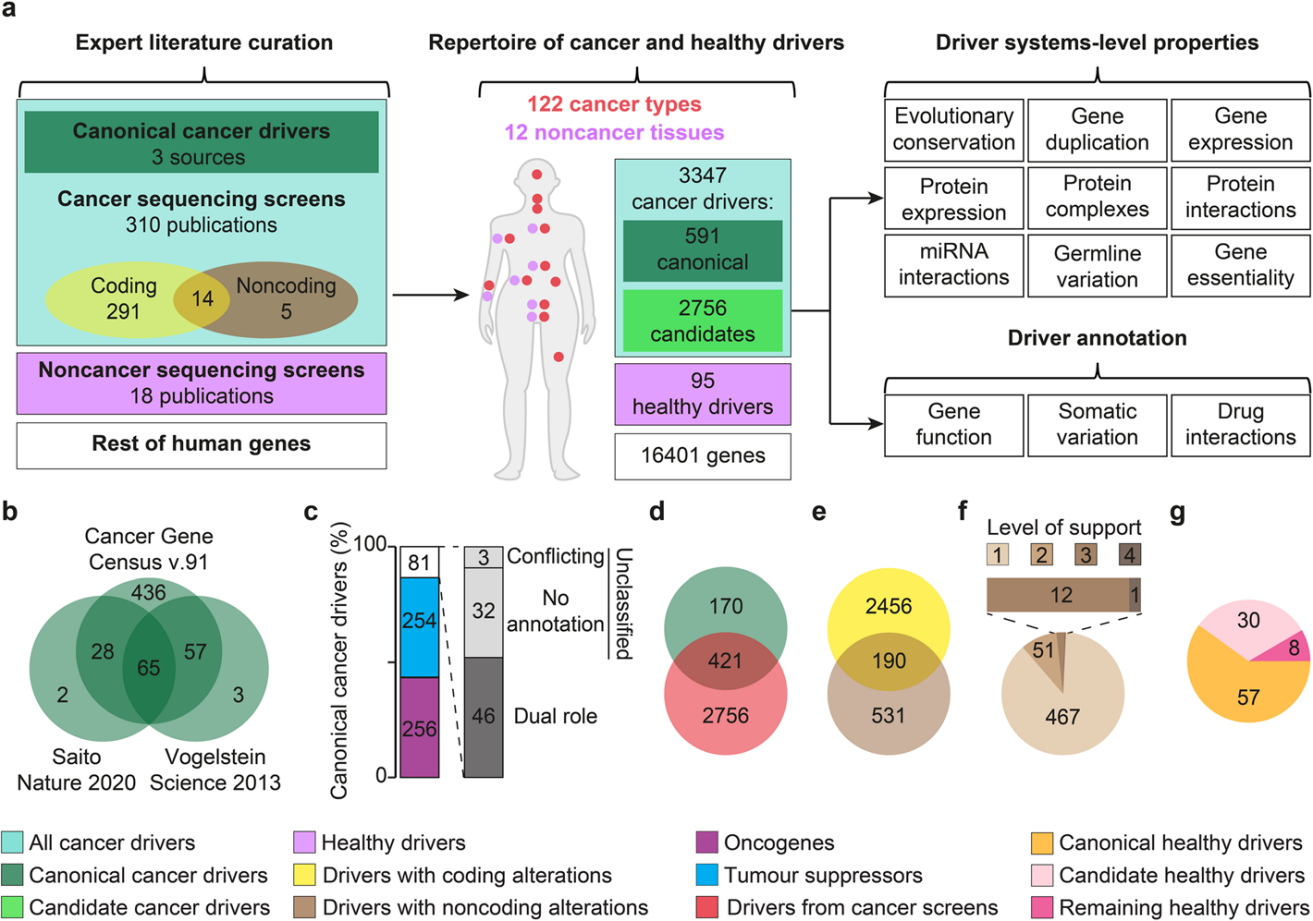
Collection of a comprehensive repertoire of cancer and healthy drivers. **a** Literature review and driver annotation workflow. Expert literature curation of 331 publications led to a repertoire of cancer and healthy drivers in a variety of cancer and non-cancer tissues. Combining multiple data sources, a set of properties and annotations was computed for all these drivers. **b** Intersection of canonical drivers from three sources [17–19] that passed our manual curation. **c** Classification of canonical cancer drivers in tumor suppressors and oncogenes. Eighty-one cancer drivers had a dual role or could not be classified. **d** Intersection of canonical and candidate driver genes from 310 sequencing screens. Genes whose driver role had only statistical support were considered candidate cancer drivers. **e** Intersection between cancer drivers with coding and non-coding alterations. **f** Level of support for the driver role of 531 cancer genes with non-coding driver alterations only. Level 1 means that the gene was predicted as a driver only in one cancer sequencing screen; levels 2, 3, and 4 mean that it was predicted by two, three, or four screens or that it had experimental support. Experimental support was gathered from the 19 publications reporting non-coding cancer drivers (Additional file 1, Table S1) and from the CNCDatabase [20] and included in vitro and in vivo experiments, modification of gene expression, and survival association. **g** Proportion of healthy drivers that are also canonical or candidate cancer drivers, classified as canonical and candidate healthy drivers, respectively

We conducted a census of currently known drivers through a comprehensive literature review of 331 scientific articles published between 2008 and 2020 describing somatically altered genes with a proven or predicted role in cancer or non-cancer somatic evolution (Fig. 1a). These publications included three sources of experimentally validated (canonical) cancer drivers, 311 sequencing screens of cancer (293) and non-cancer (18) tissues, and 17 pancancer studies (Additional file 1, Table S1). Each paper was assessed by at least two independent experts (Additional file 2, Fig. S1A-C) returning a total of 3355 drivers, 3347 in 122 cancer types and 95 in 12 non-cancer tissues, respectively (Fig. 1a). We further computed the systems-level properties of drivers and annotated their function, somatic variation, and drug interactions (Fig. 1a).

We reviewed the three sources of canonical cancer drivers [17–19] to exclude false positives (Additional file 3, Table S2) and fusion genes whose properties could not be mapped. Only 11% of the resulting 591 canonical drivers (Additional file 4, Table S3) were common to all three sources (Fig. 1b), indicating poor consensus even in well-known cancer genes. We further annotated the genetic mode of action for > 86% of canonical drivers, finding comparable proportions of oncogenes or tumor suppressors (Fig. 1c). The rest had a dual role or could not be univocally classified.

We extracted additional cancer drivers from the curation of 310 sequencing screens that applied a variety of statistical approaches (Additional file 2, Fig. S1 D) to identify cancer drivers among all altered genes. After removing possible false positives (Additional file 3, Table S2), the final list included 3177 cancer drivers, 2756 of which relied only on statistical support (candidate cancer drivers) and 421 were canonical drivers (Fig. 1d, Additional file 4, Table S3). Therefore, 170 canonical drivers have never been detected by any method, suggesting that they may elicit their role through non-mutational mechanisms or may fall below the detection limits of current approaches. Given the prevalence of cancer coding screens (Fig. 1a), only coding driver alterations have been reported for most genes (Fig. 1e) while 16% of them (531) were identified as drivers uniquely in non-coding screens. Since the prediction of drivers with non-coding alterations remains challenging, we further investigated the type of support that these genes had for their driver activity. The overwhelming majority of them (467 genes, 87%) have been predicted as drivers in only one screen. The remaining 64 genes are canonical drivers, have been predicted as drivers in multiple screens, or have additional experimental support for their driver activity (Fig. 1f).

Applying a similar approach (Additional file 2, Fig. S1 A-C), we reviewed 18 sequencing screens of healthy or diseased (non-cancer) tissues. They collectively reported 95 genes whose somatic alterations could drive non-malignant clone formation (healthy drivers). Interestingly, only eight of them were not cancer drivers (Fig. 1g, Additional file 4, Table S3), suggesting a high overlap between genetic drivers of cancer and non-cancer evolution. However, since many non-cancer screens only re-sequenced cancer genes or applied methods developed for cancer genomics (Additional file 2, Fig. S1E), this overlap may be overestimated.

### The ability to capture cancer but not healthy driver heterogeneity increases with the donor sample size

To compare cancer and healthy drivers across and within tissues, we grouped the 122 cancer types and 12 non-cancer tissues into 12 and seven organ systems, respectively (the “Methods” section).

**Fig. 2:**
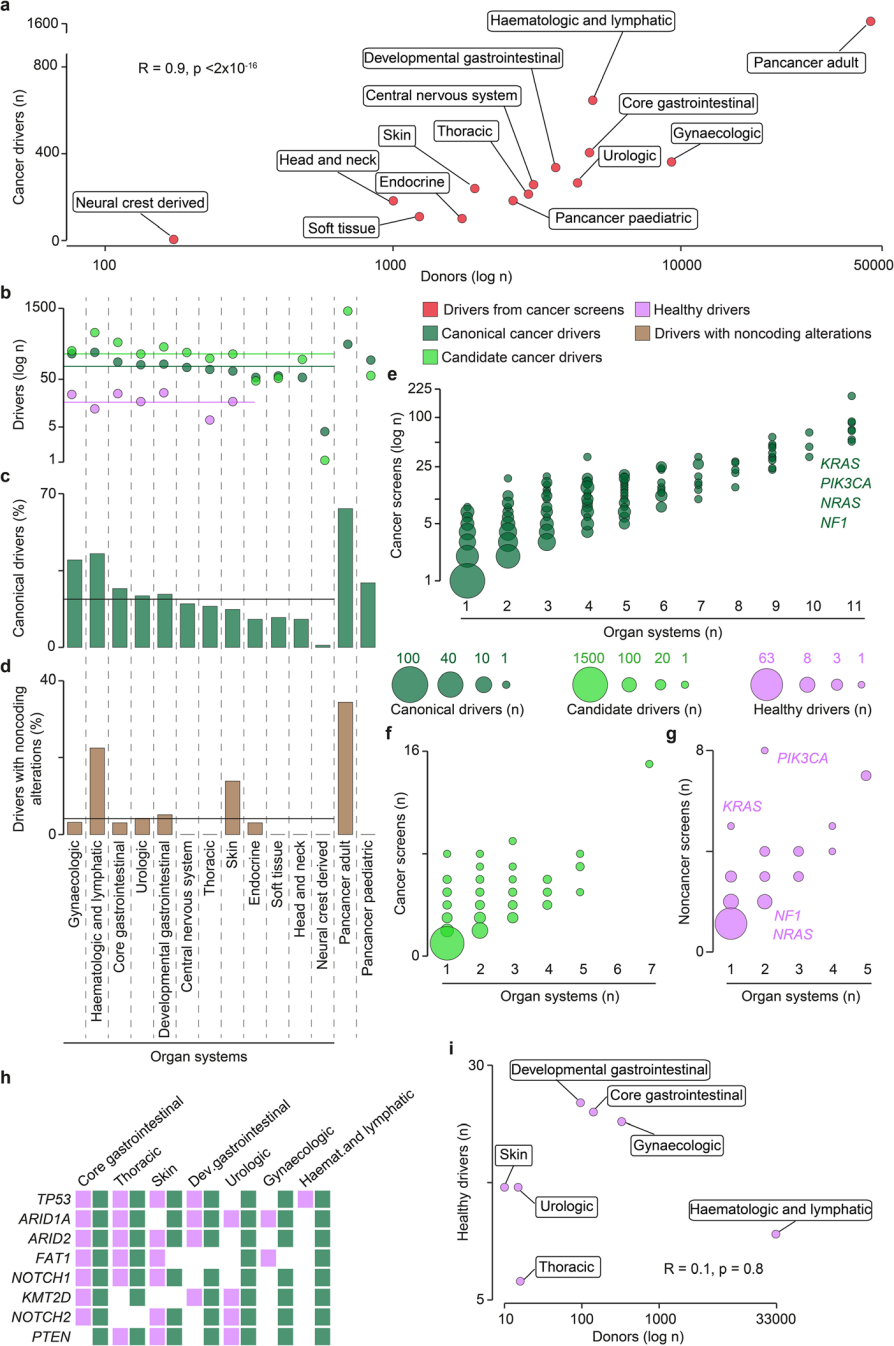
Distribution of driver annotations by organ system. **a** Correlation between numbers of sequenced donors and identified cancer drivers across organ systems. Spearman correlation coefficient *R* and associated *p*-value are shown. **b** Number of canonical, candidate, and healthy drivers in each organ system. Horizontal lines indicate the median number of canonical (92), candidate (160), and healthy (17) drivers across organ systems. **c** Proportion of canonical drivers detected in each organ system over canonical drivers detected in all cancer screens (421). The horizontal line indicates the median across all organ systems (22%). **d** Proportion of genes with non-coding driver alterations over all cancer drivers in each organ system. The horizontal line indicates the median across all organ systems (4%). Number of canonical (**e**), candidate (**f**), and healthy (**g**) drivers across screens and organ systems. Representative genes with different recurrence between cancer and healthy tissues are indicated. **h** Organ system distribution of the top eight recurrent healthy drivers. The full list is provided as Additional file 6, Table S5. **i** Correlation between numbers of sequenced donors and identified healthy drivers across organ systems. Spearman correlation coefficient *R* and associated *p*-value are shown

Despite the high numbers of sequenced samples (Additional file 5, Table S4) and detected drivers (Fig. 1), several lines of evidence indicated that our knowledge of cancer drivers is still incomplete. First, we detected a strong positive correlation between cancer drivers and donors overall (Fig. 2a) and in individual organ systems (Additional file 2, Fig. S2). This suggests that the current ability to identify new drivers depends on the number of samples included in the analysis. Second, candidates outnumbered canonical drivers in all organ systems except those with a small sample size or low mutation rate such as pediatric cancers, where only the most recurrent canonical drivers could be identified (Fig. 2b). Third, large donor cohorts enabled the detection of a broader representation of canonical drivers than small cohorts (Fig. 2c). For example, pooling thousands of samples together led to > 60% of canonical drivers being detected in adult pancancer re-analyses. Therefore, the size of the cohort influences the level of completeness and heterogeneity of the cancer driver repertoire. This is not surprising since all current approaches act at the cohort level, searching for positively selected genes altered more frequently than expected (Additional file2, Fig. S1D).

Our analysis also showed that the contribution of non-coding driver alterations remains largely unappreciated and non-coding drivers have not yet been reported in several tumors, including all pediatric cancers (Fig. 2d). Owing to the re-analysis of large whole-genome collections [21–26], almost 40% of adult pancancer drivers were instead modified by non-coding alterations (Fig. 2d). Hematologic and skin tumors also had a high proportion of non-coding driver variants thanks to screens focused on non-coding mutations [27, 28]. Therefore, the re-analysis of already available whole-genome data and further sequencing screens of non-coding variants are needed to fully appreciate their driver contribution.

Compared to cancer, sequencing screens of non-cancer tissues are still in their infancy, as reflected by the lower numbers of screened tissues and detected drivers (Fig. 2b). Despite this, some similarities and differences with cancer drivers could already be observed. Like cancer drivers (Fig. 2e, f, Additional file 6, Table S5), also healthy drivers were mostly organ-specific (Fig. 2g) and the most recurrent healthy drivers were also cancer drivers in the same organ system (Fig. 2h, Additional file 6, Table S5). However, some recurrent cancer drivers (*KRAS*, *PI3KCA*, *NRAS*, *NF1*) were reported to drive non-cancer clonal expansion only in one or two organ systems (Fig. 2g). Therefore, differences start to emerge at the tissue level between drivers of cancer and non-cancer evolution. Moreover, unlike cancer drivers, no correlation existed between the numbers of drivers and donors (Fig. 2i). This is likely affected by the lower number of non-cancer sequencing studies available so far. If additional studies will confirm the absence of correlation, this may indicate that the healthy driver repertoire is easier to saturate since fewer drivers are needed to initiate and sustain non-cancer clonal expansion [10, 11].

### Alteration pattern hints at driver mode of action and confirms the incompleteness of the driver repertoire

**Fig. 3:**
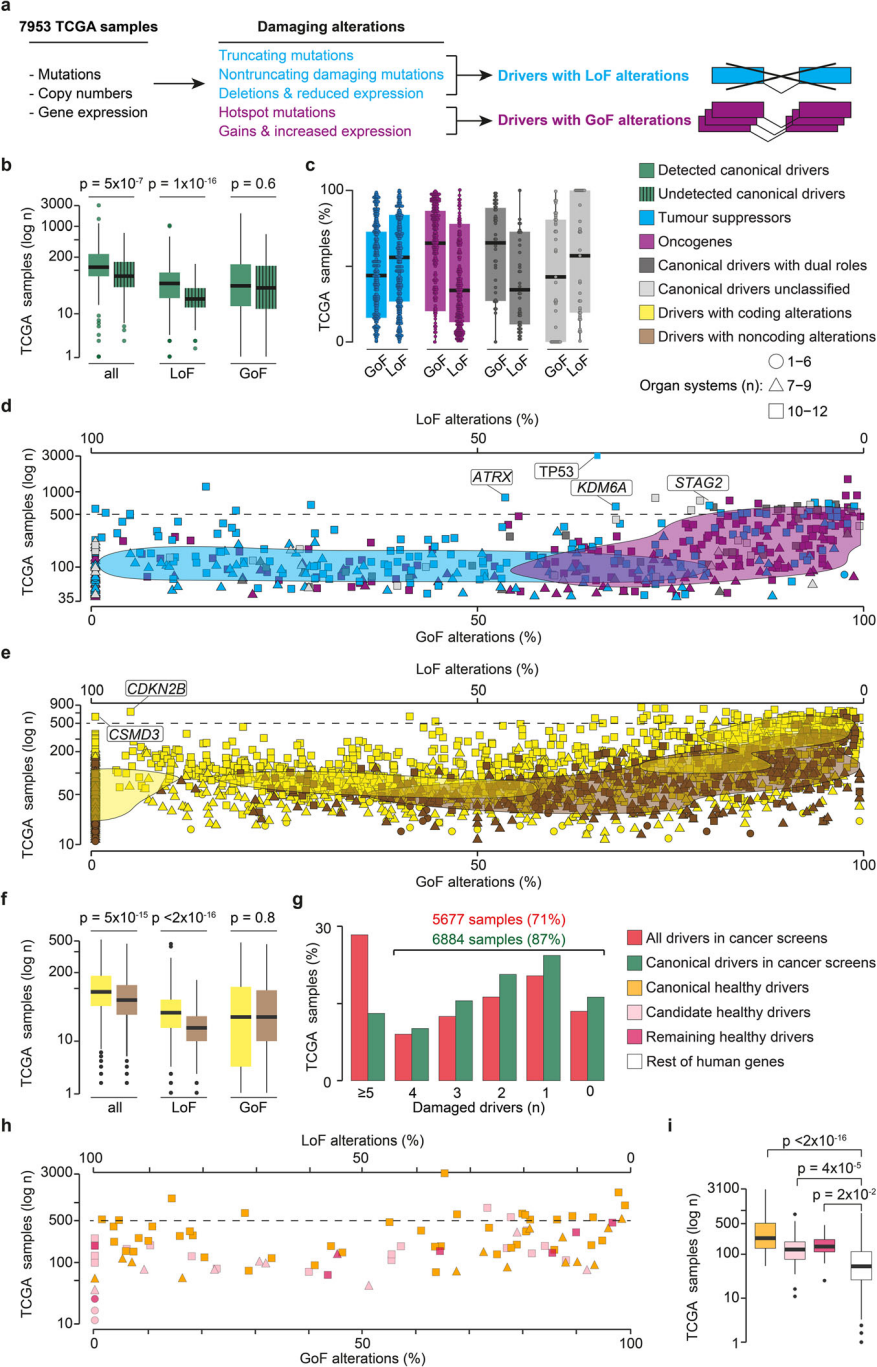
Damaging alteration pattern of drivers in TCGA. **a** Identification of damaged drivers in 7953 TCGA samples. Mutations, gene deletions, and amplifications were annotated according to their predicted damaging effect. This allowed to distinguish drivers acquiring loss-of-function (LoF) or gain-of-function (GoF) alterations. **b** Number of TCGA samples with damaging alterations (all, LoF, GoF) in canonical drivers that were detected (421) or undetected (170) by cancer driver detection methods. **c** Proportion of TCGA samples with GoF and LoF alterations in tumor suppressors, oncogenes, and canonical drivers with a dual or unclassified role. Proportion of TCGA samples with GoF and LoF alterations in (**d**) canonical drivers and (**e**) candidate drivers. Genes mentioned in the text are highlighted. The two-dimensional Gaussian kernel density estimations were calculated for each driver group using the R density function. **f** Number of TCGA samples with damaging alterations (all, LoF, GoF) in drivers previously reported in coding and non-coding sequences. **g** Proportion of samples with variable numbers of all damaged drivers or only canonical drivers. **h** Proportion of TCGA samples with GoF and LoF alterations in healthy drivers. Canonical and candidate healthy drivers correspond to genes with a known or predicted cancer driver role. **i** Number of TCGA samples with damaged canonical, candidate, and remaining healthy drivers and the rest of human genes. All distributions were compared using a two-sided Wilcoxon rank-sum test

To gain further insights into their mode of action, we mapped the type of alterations acquired by cancer and healthy drivers in 34 cancer types from TCGA. After predicting the damaging alterations in 7953 TCGA samples with matched mutation, copy number, and gene expression data (the “Methods” section), we identified the drivers with loss-of-function (LoF) and gain-of-function (GoF) alterations in these samples, respectively (Fig. 3a).

The comparison between canonical cancer drivers detected and undetected in sequencing screens (Fig. 1d) revealed that the latter were damaged in a significantly lower number of samples, due to fewer LoF alterations (Fig. 3b, Additional file 2, Fig. S3A). GoF alterations were instead comparable between the two groups, suggesting that current driver detection methods fail to identify drivers that undergo copy number gains but are rarely mutated.

We confirmed that the driver alteration patterns reflected their mode of action, with canonical tumor suppressors and oncogenes showing a prevalence of LoF and GoF alterations, respectively (Fig. 3c). Canonical drivers with a dual role resembled the alteration pattern of oncogenes while those still unclassified had a prevalence of LoF alterations, suggesting a putative tumor suppressor role (Fig. 3c). While all frequently altered (> 500 samples) oncogenes were overwhelmingly modified by GoF alterations (Additional file 7, Table S6), 16 of the 22 most frequently altered tumor suppressors had a prevalence of GoF alterations (Fig. 3d). In the majority of cases, this was due to different alteration patterns across organ systems (Additional file 2, Fig. S3B), and a possible oncogenic role has been documented for some others [29–38].

Since candidate drivers had no annotation of their mode of action, we reasoned that their alteration pattern could hint at their role as tumor suppressors or oncogenes. According to their prevalent pancancer alterations, 1318 candidates could be classified as putative tumor suppressors and 1405 as putative oncogenes (Additional file 7, Table S6). Interestingly, while candidates with predicted coding driver alterations showed similar distributions of LoF and GoF alterations (Fig. 3e), those with only non-coding driver alterations had a significantly lower occurrence of LoF alterations (Fig. 3f, Additional file 2, Fig. S3C). This may suggest an activating role for their non-coding alterations too. Almost all candidates damaged in ≥ 500 samples (111/115) were putative oncogenes (Fig. 3e, Additional file 7, Table S6). Of the four putative tumor suppressors, *CSMD3* has a disputed cancer role [39–41] and a likely inflated mutation rate [42], while *CDKN2B* cooperates with its paralog *CDKN2A* to inhibit cell cycle [43], supporting its tumor suppressor role.

The number of damaged cancer drivers in individual TCGA samples confirmed that, despite all efforts, the current driver repertoire is still largely incomplete. The large majority of samples (71% and 87%, considering all drivers or only canonical drivers, respectively) had less than five damaged drivers, and ~ 15% of them had no damaged driver (Fig. 3g).

Given their high overlap with cancer drivers, most healthy drivers were recurrently damaged in cancer samples with no prevalence of GoF or LoF alterations (Fig. 3h, Additional file 7, Table S6). Interestingly, all healthy drivers, even the eight with no cancer involvement, were damaged in significantly more cancer samples than the rest of human genes (Fig. 3i). Moreover, 57% of TCGA samples had at least two altered drivers, one of which was a healthy driver, further supporting the hypothesis that more than one driver may be needed to promote the transformation of non-malignant clones into cancer [10, 11].

### Properties of cancer and healthy drivers support their central role in the cell

A substantial body of work including our own [44–53] has shown that cancer drivers differ from the rest of the genes for an array of systems-level properties (Fig. 1a) that are a consequence of their unique evolutionary path and role in the cell. Using our granular annotation of drivers, we set out to check for similarities and differences across the driver groups.

**Fig. 4:**
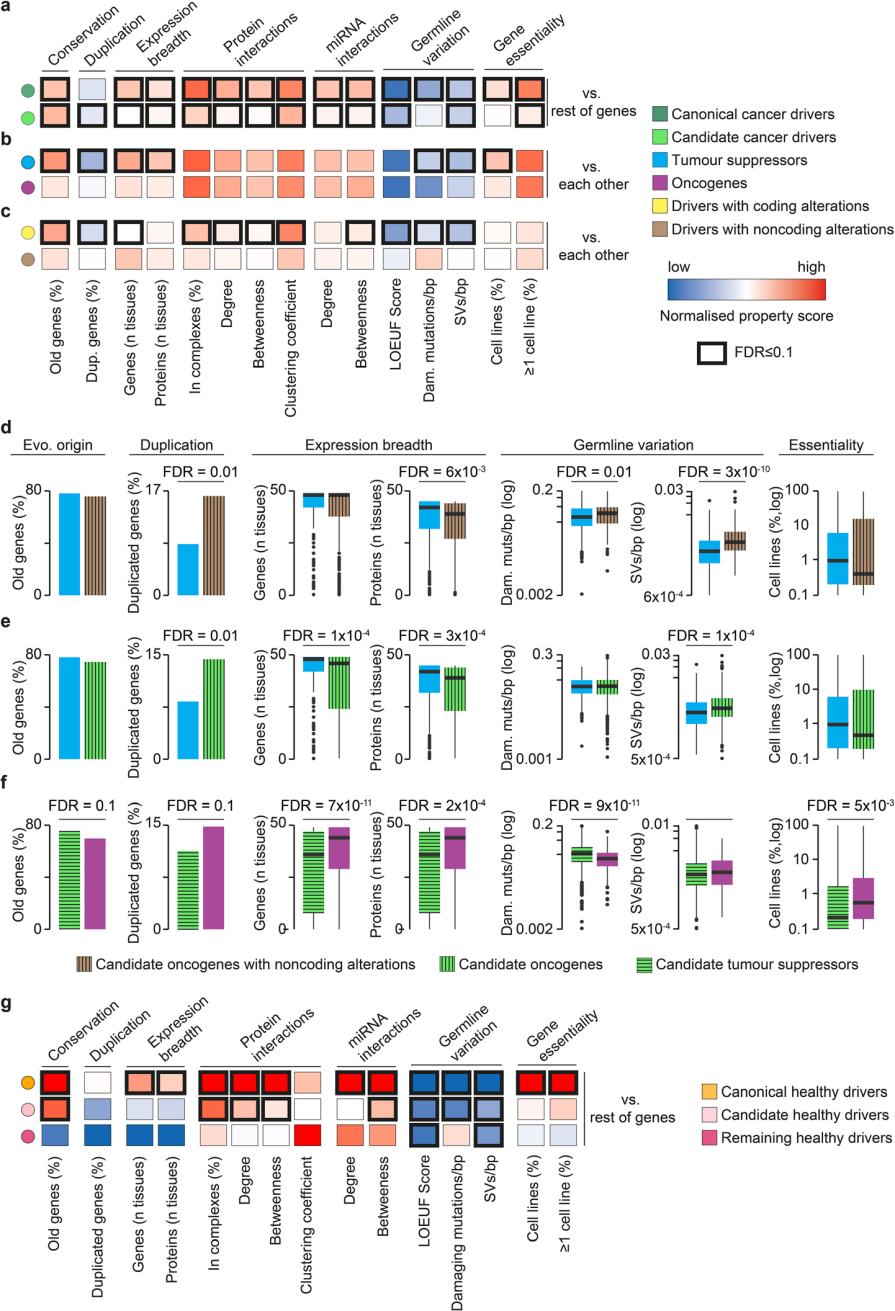
Systems-level properties of cancer and healthy drivers. Comparisons of systems-level properties between (**a**) canonical or candidate cancer drivers and the rest of human genes, (**b**) tumor suppressors and oncogenes, and (**c**) cancer genes with coding driver alterations and cancer genes with non-coding driver alterations. The normalized property score was calculated as the normalized difference between the median (continuous properties) or proportion (categorical properties) values in each driver group and the rest of human genes (the “Methods” section). Comparisons of systems-level properties between (**d**) candidate oncogenes with non-coding driver alterations (324) and canonical tumor suppressors, (**e**) candidate oncogenes (1405) and canonical tumor suppressors, and (**f**) candidate tumor suppressors (1318) and canonical oncogenes. **g**. Comparisons of systems-level properties between canonical healthy, candidate healthy, and remaining healthy drivers and the rest of human genes. Proportions of old (pre-metazoan), duplicated, essential genes, and proteins involved in the complexes were compared using a two-sided Fisher’s exact test. Distributions of gene and protein expression, protein-protein, miRNA-gene interactions, and germline variation were compared using a two-sided Wilcoxon rank-sum test. False discovery rate (FDR) was corrected for using Benjamini-Hochberg

We confirmed that cancer drivers, and in particular canonical drivers, were more conserved throughout evolution and less likely to retain gene duplicates than other human genes (Fig. 4a, Additional file 8, Table S7). They also showed broader tissue expression, engaged in a larger number of protein complexes, and occupied more central and highly connected positions in the protein-protein and miRNA-gene networks (Fig. 4a). We reported substantial differences between tumor suppressors and oncogenes, with the former enriched in old and single-copy genes showing broader tissue expression (Fig. 4b, Additional file 8, Table S7).

We further expanded the systems-level properties of cancer drivers by exploring their tolerance towards germline variation, because this may indicate their essentiality. Using germline data from healthy individuals [54], we compared the loss-of-function observed/expected upper bound fraction (LOEUF) score, which quantifies selection towards LoF variation [54] as well as the number of damaging mutations and structural variants (SVs) per coding base pairs (bp) between drivers and the rest of genes (the “Methods” section). Cancer drivers, and in particular canonical drivers, had a significantly lower LOEUF score and retained fewer damaging germline mutations and SVs than the rest of the genes (Fig. 4a). This indicates that they are indispensable for cell survival in the germline. Selection against harmful variation was stronger in tumor suppressors than oncogenes (Fig. 4b). This was supported by a significantly higher proportion of cell lines where cancer drivers, and in particular tumor suppressors, were essential (Fig. 4a, b), as gathered from the integration of nine genome-wide essentiality screens [55–63] (the “Methods” section).

Genes with non-coding driver alterations had weaker systems-level properties than those with coding alterations (Fig. 4c, Additional file 8, Table S7) and the subset of them with > 50% GoF alterations resembled the property profile of oncogenes when compared to tumor suppressors (Fig. 4d, Additional file 8, Table S7). In general, all candidate drivers with a prevalence of GoF were similar to oncogenes, showing a higher proportion of duplicated genes, narrower tissue expression, and higher tolerance to germline variation than tumor suppressors (Fig. 4e, Additional file 8, Table S7). Conversely, candidate drivers with a prevalence of LoF were older, less duplicated, and less tolerant to germline variation than oncogenes (Fig. 4f, Additional file 8, Table S7).

Systems-level properties of healthy drivers varied according to the overlap with cancer drivers (Fig. 4g, Additional file 8, Table S7). Intriguingly, canonical healthy drivers showed stronger systems-level properties than any other group of drivers. In particular, they were enriched in evolutionarily conserved and broadly expressed genes encoding highly inter-connected proteins are regulated by many miRNAs. Moreover, these genes showed a strong selection against germline variation and high enrichment in essential genes (Fig. 4g). They therefore represent a core of genes with a very central role in the cell, whose modifications are not tolerated in the germline but are selected for in somatic cells because they confer selective growth advantages. Candidate healthy drivers and those not involved in cancer had a substantially different property profile (Fig. 4g). Although numbers are too low for any robust conclusion, it is tempting to speculate that genes able to initiate non-cancer clonal expansion but not tumorigenesis may follow a different evolutionary path.

### The Network of Cancer Genes: an open-access repository of annotated drivers

**Fig. 5:**
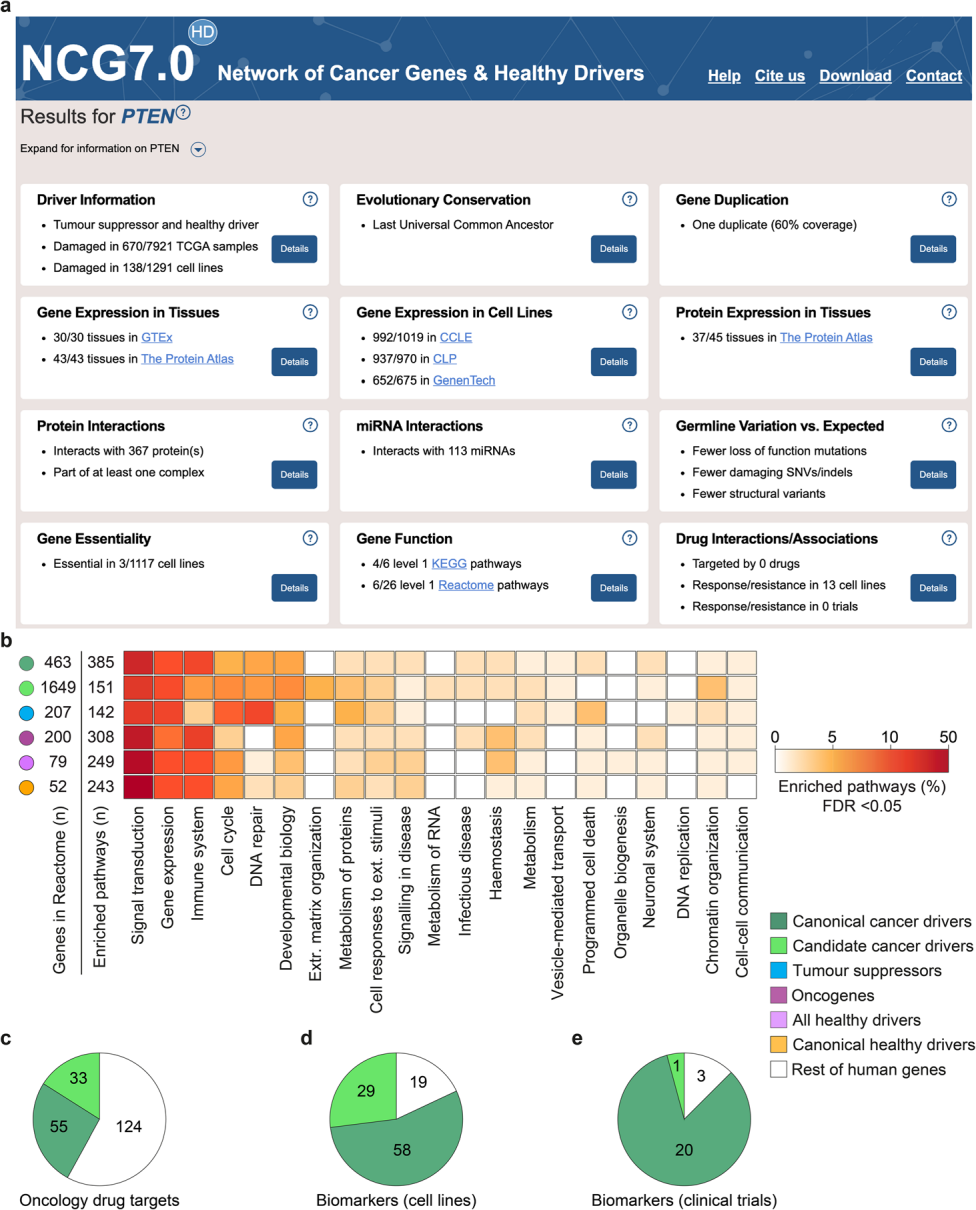
NCG^HD^ annotations of driver genes. **a** Example of the type of annotation provided in NCG^HD^ for cancer and healthy drivers (in this case *PTEN*). Annotation boxes can be expanded for further details, with the possibility of intersecting data interactively (for example, in the case of protein-protein or miRNA-gene interactions) and downloading data for local use. **b** Proportion of Reactome levels 2–8 enriched pathways mapping to the respective level 1 in each driver group. Enrichment was measured comparing the proportion of drivers in each pathway to that of the rest of human genes with a one-sided Fisher’s exact test. FDR was calculated using Benjamini-Hochberg. The numbers of drivers and enriched Reactome pathways are reported for each group. Proportion of canonical and candidate cancer divers and rest of genes that are (**c**) targets of FDA-approved antineoplastic drugs or biomarkers of response or resistance to oncological drugs in (**d**) cancer cell lines and (**e**) clinical studies. The corresponding numbers for each group are also shown

We collected the whole repertoire of 3347 cancer and 95 healthy drivers, their literature support, and properties in the seventh release of the Network of Cancer Genes and Healthy Drivers (NCG^HD^) database. NCG^HD^ is accessible through an open-access portal that enables interactive queries of drivers (Fig. 5a) as well as the bulk download of the database content.

In addition to the known or predicted mode of action and systems-level properties of cancer and healthy drivers, NCG^HD^ 7.0 also annotates their function, alteration pattern, and gene expression profile in TCGA and cancer cell lines, reported interactions with antineoplastic drugs, and potential role as treatment biomarkers (Fig. 5b). Altogether, this constitutes an extensive compendium of annotation of driver genes, including information relevant for planning experiments involving them.

Functional gene set enrichment analysis showed that at least 60% of enriched pathways (FDR < 0.05) in any driver group converge to five broad functional processes (signal transduction, gene expression, immune system, cell cycle, and DNA repair, Fig. 5b, Additional file 9, Table S8). Within these, tumor suppressors showed a prevalence in cell cycle and DNA repair pathways, while oncogenes were enriched in the gene expression and immune system-related pathways (Additional file 9, Table S8). Healthy drivers closely resembled the functional profile of cancer drivers, given the high overlap (Fig. 5b). Because of the low number, it was not possible to assess the functional enrichment of healthy drivers not involved in cancer.

More than 9% of canonical cancer drivers are targets of anti-cancer drugs and cancer drivers constitute around 40% of their targets (Fig. 5c). Moreover, most of the genes used as biomarkers of resistance or response to treatment in cell lines (Fig. 5d) or clinical trials (Fig. 5e) are cancer drivers, with an overwhelming prevalence of canonical cancer drivers.

## Discussion

The wealth of cancer genomic data and the availability of increasingly sophisticated analytical approaches for their interpretation have substantially improved the understanding of how cancer starts and develops. However, our in-depth analysis of the vast repertoire of drivers that have been collected so far shows clear limits in the current knowledge of the driver landscape.

The identification of drivers as genes under positive selection or with a higher than expected mutation frequency within a cohort of patients has biased the current cancer driver repertoire towards genes whose coding point mutations or small indels frequently recur across patients. This strongly impairs the ability to map the full extent of driver heterogeneity leading to an underappreciation of the driver contribution of rarely altered genes and those modified through non-coding or gene copy number alterations, particularly amplifications. It also results in a sizeable fraction of samples with very few or no cancer drivers. This gap can be solved by complementing cohort-level approaches with methods that account for all types of alterations and predict drivers in individual samples, for example identifying their network deregulations [64–66] or applying machine learning to identify driver alterations [67]. Alternatively, we have shown that systems-level properties capture the main features of cancer drivers, justifying their use for patient-level driver detection [68, 69].

Our comprehensive study has also shown that cancer sequencing screens have so far mostly focused on resequencing and analyzing the protein-coding portion of cancer genomes, leaving the contribution of non-coding drivers mostly uncovered. This bias may be addressed by performing additional cancer whole genome sequencing screens and improving analytical methods for the prediction of non-coding driver alterations.

Biases are starting to emerge also in the knowledge of healthy drivers. Many non-cancer sequencing screens only targeted cancer genes and healthy driver detection methods used so far were originally developed for cancer genomics. Both these factors may contribute at least in part to explain the high overlap between drivers of cancer and non-cancer evolution. An unbiased investigation of altered genes able to promote clonal expansion but not tumorigenesis could confirm whether their properties are indeed different from cancer drivers as suggested by our initial analysis on the few of them that have been identified so far. Additionally, the investigation of somatically mutated clones in non-cancer tissues has just started and new screens are continuously published. The integrated analysis of these new studies will broaden our understanding of non-cancer clonal expansion and further clarify its relationship with cancer transformation.

Our literature review did not cover driver genes deriving from chromosomal rearrangements or epigenetic changes because of their scattered annotations in the literature and difficulty in mapping their properties. Adding these genes to the repertoire when their knowledge will be mature will help close the gaps in the knowledge of the genetic drivers of tumorigenesis.

## Conclusions

Our comprehensive analysis of cancer sequencing screens showed that the current repertoire of cancer driver genes is still incomplete and biased towards frequent mutations altering the gene coding sequence. This calls for the need for additional screens and methods to identify further coding and non-coding cancer drivers at single patient resolution. We confirmed the central role of cancer drivers within the cell, which is reflected in their evolutionary path and is shared by the majority of known healthy drivers. Further sequencing screens of healthy tissues are needed to clarify whether this is a feature of all genes whose mutations can driver non-cancer clonal expansion or there is a group of healthy drivers that underwent a different evolutionary path.

## Methods

### Literature curation

A literature search was carried out in PubMed, TCGA (https://www.cancer.gov/tcga) and ICGC (https://dcc.icgc.org/) to retrieve cancer screens published between 2018 and 2020 (Additional file 2, Fig. S1A). This resulted in 135 coding and 154 non-coding cancer screens. Of these, only 80 and 37 were retained after examining abstracts and full text, respectively. Criteria for removal were the absence of driver genes or driver detection methods and the impossibility to map non-coding driver alterations to genes. The 37 new cancer screens were added to 273 publications previously curated by our team [70], totaling 310 publications (Additional file 1, Table S1). A similar literature search retrieved 24 sequencing screens of non-cancer tissues publications, 18 of which were retained after the abstract and full-text examination (Additional file 2, Fig. S1A; Additional file 1, Table S1). Each paper was reviewed independently by two experts and further discussed if annotations differed to extract the list of driver genes, the number of donors, the type of screen (whole-genome, whole-exome, target gene re-sequencing), the cancer or non-cancer tissues, and the driver detection method (Additional file 2, Fig. S1B).

Canonical cancer drivers were extracted from two publications [17, 18] and the Cancer Gene Census [71] v.91. In the latter case, all tiers 1 and 2 genes were retained, except those from genomic rearrangements leading to gene fusion (Additional file 2, Fig. S1B). Collected genes were further classified as tumor suppressor, oncogene, or having a dual role according to the annotation in the majority of sources. Genes with conflicting or unavailable annotation were left unclassified.

Drivers from cancer screens and canonical sources underwent further filtering (Additional file 2, Fig. S1C). First, they were intersected with a list of 148 possible false positives [18, 42]. After a manual check of the supporting evidence, two drivers were retained as canonical, five were considered as candidates, and 41 were removed (Additional file 3, Table S2). The three resulting lists (canonical drivers, drivers from cancer screens, and healthy drivers) were intersected to annotate canonical drivers in cancer screens, remaining drivers in cancer screens (candidate cancer drivers), canonical healthy drivers, candidate healthy drivers, and remaining healthy drivers (Additional file 2, Fig. S1C; Additional file 4, Table S3).

Cancer types and non-cancer tissues were mapped to organ systems using previous classification [72]. Cancer types not included in this classification were mapped based on their histopathology (retinoblastoma to central nervous system, vascular and peripheral nervous system cancers to soft tissue, penile tumors to urologic system).

### Pancancer TCGA data

A dataset of 7953 TCGA samples with quality-controlled mutation (SNVs and indels), copy number, and gene expression data in 34 cancer types was assembled from the Genomic Data Commons portal I [73] (https://portal.gdc.cancer.gov/). Mutations were annotated with ANNOVAR [74] (April 2018) and dbNSFP [75] v3.0 and only those identified as exonic or splicing were retained. Damaging mutations included (1) truncating (stopgain, stoploss, frameshift) mutations, (2) missense mutations predicted by at least seven out of 10 predictors (SIFT [76], PolyPhen-2 HDIV [77], PolyPhen-2 HVAR, MutationTaster [78], MutationAssessor [79], LRT [80], FATHMM [81], PhyloP [82], GERP++RS [83], and SiPhy [84]), (3) splicing mutations predicted by at least one of two splicing-specific methods (ADA [75] and RF [75]), and (4) hotspot mutations identified with OncodriveCLUST [85] v1.0.0.

Copy number variant (CNV) segments, sample ploidy, and sample purity values were obtained from TCGA SNP arrays using ASCAT [86] v.2.5.2. Segments were intersected with the exonic coordinates of 19,756 human genes in hg19 and genes were considered to have CNV if at least 25% of their transcribed length was covered by a CNV segment. RNA-Seq data were used to filter out false-positive CNVs. Putative gene gains were defined as copy number (CN) > 2 times sample ploidy and the levels of expression were compared between samples with and without each gene gain using a two-sided Wilcoxon rank-sum test and corrected for multiple testing using Benjamini-Hochberg. Only gene gains with a false discovery rate (FDR) < 0.05 were retained. Homozygous gene losses had CN = 0 and fragments per kilobase per million (FPKM) values < 1 over sample purity. Heterozygous gene losses had CN = 1 or CN = 0 but FPKM values > 1 over sample purity. This resulted in 2,192,832 redundant genes damaged in 7921 TCGA samples.

In total, 518,115 genes were considered to acquire LoF alterations because they underwent homozygous deletion or had truncating, missense damaging, splicing mutations, or double hits (CN = 1 and LoF damaging mutation), while 1,674,717 genes were considered to acquire GoF alterations because they had a hotspot mutation or underwent gene gain with increased expression (Fig. 3a).

### Systems-level properties

Protein sequences from RefSeq [87] v.99 were aligned to hg38 using BLAT [88]. Unique genomic loci were identified for 19,756 genes based on gene coverage, span, score, and identity [89]. Genes sharing at least 60% of their protein sequence were considered as duplicates [46].

Evolutionary conservation was assessed for 18,922 human genes using their orthologs in EggNOG [90] v.5.0. Genes were considered to have a pre-metazoan origin (and therefore conserved in evolution) if they had orthologs in prokaryotes, eukaryotes, or opisthokonts [53].

Gene expression for 19,231 genes in 49 healthy tissues was derived from the union of Protein Atlas [91] v.19.3 and GTEx [92] v.8. Genes were considered to be expressed in a tissue if their expression value was ≥ 1 transcript per million (TPM). Protein expression for 13,229 proteins in 45 healthy tissues was derived from Protein Atlas [91] v.19.3 retaining the highest value when multiple expression values were available.

A total of 542,397 non-redundant binary interactions between 17,883 proteins were gathered from the integration of five sources (BioGRID [93] v.3.5.185, IntAct [94] v.4.2.14, DIP [95] (February 2018), HPRD [96] v.9 and Bioplex [97] v.3.0). Data on 9476 protein complexes involving 8504 proteins were derived from CORUM [98] v.3.0, HPRD [96] v.9 and Reactome [99] v.72. Experimentally supported interactions between 14,747 genes and 1758 miRNAs were acquired from miRTarBase [100] v.8.0 and miRecords [101] v.4.0. Degree, betweenness, and clustering coefficient were calculated for protein and miRNA networks using the igraph R package [102] v.1.2.6.

The loss-of-function observed/expected upper bound fraction (LOEUF) score for 18,392 genes was obtained from gnomAD [54] v.2.1.1. Germline mutations (SNVs and indels) were obtained from the union of 2504 samples from the 1000 Genomes Project Phase 3 [103] v.5a and 125,748 samples from gnomAD [54] v.2.1.1. Mutations were annotated with ANNOVAR [74] (October 2019), and 18,812 genes were considered as damaged using the same definitions as for TCGA samples. A total of 32,558 germline SVs for 14,158 genes were derived using 15,708 samples from gnomAD [54] v.2.1.1. The numbers of damaging mutations and SVs per base pairs (bp) were calculated for each gene.

Essentiality data for 19,013 genes in 1122 cell lines were obtained integrating three RNAi knockdown and six CRISPR Cas9 knockout screens [55–63]. Genes with CERES [57] or DEMETER [63] scores < − 1 or Bayes score [104] > 5 were considered as essential.

Proportions of duplicated, pre-metazoan, essential genes, and proteins engaging in complexes were compared between the gene groups using two-sided Fisher’s exact test. Distributions of tissues where genes or proteins were expressed, protein and miRNA network properties, LOEUF scores, damaging mutations, and SVs per bp were compared between the gene groups using a two-sided Wilcoxon test. Multiple comparisons within each property were corrected using Benjamini-Hochberg. For each systems-level property in each driver group (*d*), a normalized property score was calculated as:

$$ \mathrm{Normalised}\ \mathrm{property}\ \mathrm{score}=\operatorname{sgn}\left({\Delta}_d\right)\times \frac{\left|{\Delta}_d\right|-\underset{t}{\min}\left|{\Delta}_t\right|}{\underset{t}{\max}\left|{\Delta}_{\mathrm{t}}\right|-\underset{t}{\min}\left|{\Delta}_t\right|} $$

where *t* represents 11 gene groups (canonical drivers, candidate drivers, tumor suppressors, oncogenes, drivers with coding alterations, drivers with non-coding alterations, canonical healthy drivers, candidate healthy drivers, remaining healthy drivers, and the rest of human genes); sgn(Δ_*d*_) is the sign of the difference; and Δ_*d*_ indicates the difference of medians (continuous properties) or proportions (categorical properties) between each driver group and the rest of human genes. Minima and maxima were taken over all 11 gene groups for each property.

### Pancancer cell line data

Mutation, CNV and gene expression data for 1291 cell lines were obtained from DepMap [56, 105] v. 20Q3. Mutations were functionally annotated using ANNOVAR [74] and LoF mutations were identified as described for TCGA samples. Hotspot mutations were detected using hotspot positions derived from TCGA. Homozygous gene deletions were defined as CN < 0.25 times cell line ploidy and expression < 1 TPM; heterozygous gene deletions were defined as 0.25 < CN < 0.75 times cell line ploidy; gene gains were defined as CN > 2 times cell line ploidy and significantly higher expression relative to cell lines with no gene gains. Genes with LoF or GoF alterations were defined as for TCGA samples. To map cell lines to organ systems, they were first associated with the TCGA cancer types and then the same classification as for TCGA was used [72].

### Driver functional annotation

Gene functions were collected for 11,778 proteins from Reactome [99] v.72 and KEGG [106] v.94.1 (levels 1 and 2). Driver enrichment in Reactome pathways (levels 2–8) compared to the rest of human genes was assessed using a one-sided Fisher’s exact test and corrected for multiple testing with Benjamini-Hochberg. Enriched pathways were then mapped to the corresponding Reactome level 1.

### Drug interactions

A total of 247 FDA-approved, antineoplastic, and immunomodulating drugs targeting 212 human genes were downloaded from DrugBank [107] v.5.1.8. Genetic biomarkers of response and resistance to drugs in cancer cell lines were obtained from Genomics of Drug Sensitivity in Cancer (GDSC) [108] v.8.2. Of those, only 467 associations with FDR ≤ 0.25 involving 129 drugs and 106 genes were retained. Genetic biomarkers of response and resistance in clinical studies were obtained from the Variant Interpretation for Cancer Consortium Meta-Knowledgebase [109] v.1. A total of 868 associations between drugs and genomic features involving 64 anti-cancer drugs and drug combinations and 24 human genes were retained [109].

### Database and website implementation

All annotations of driver genes were entered into a relational database based on MySQL [110] v.8.0.21 connected to a web interface enabling interactive retrieval of information through gene identifiers. The frontend was developed with PHP [111] v.7.4.15. The interactive displays of miRNA-gene and protein-protein interactions were implemented with the R packages Shiny [112] v.1.6.0 and igraph [102] v.1.2.6 and ran on Shiny Server v1.5.16.958.

## Supplementary Information

**Additional file 1: Table S1.** Publications describing driver genes.

**Additional file 2: Figure S1.** Literature search, review and annotation workflow; **Figure S2.** Correlation between numbers of donors and cancer drivers in individual organ systems; **Figure S3.** Patterns of driver damaging alterations in TCGA samples.

**Additional file 3: Table S2.** Putative false positive cancer drivers.

**Additional file 4: Table S3.** Canonical cancer drivers.

**Additional file 5: Table S4.** Donors in cancer and noncancer sequencing screens.

**Additional file 6: Table S5.** Drivers reported in cancer and non-cancer screens.

**Additional file 7: Table S6.** Cancer and non-cancer drivers damaged in TCGA.

**Additional file 8: Table S7.** Systems-level properties of driver genes.

**Additional file 9: Table S8.** Proportion of enriched pathways across driver groups.

**Additional file 10.** Review history.

## Data Availability

The whole content of NCG^HD^ can be freely downloaded from the website (http://network-cancer-genes.org/). No license is required. Original data were obtained from the following online sources: 1000 Genomes Project Phase 3 [103] v.5a: https://www.internationalgenome.org/category/phase-3/ BioGRID [93] v.3.5.185: https://thebiogrid.org/ Bioplex [97] v.3.0: https://bioplex.hms.harvard.edu/interactions.php CORUM [98] v.3.0: http://mips.helmholtz-muenchen.de/corum/ Depmap [59, 60] v20Q3: https://depmap.org/portal/ DIP [95] (February 2018): https://dip.doe-mbi.ucla.edu/dip/Main.cgi DrugBank [107] v.5.1.8: https://go.drugbank.com/ EggNog [90] v.5: http://eggnog5.embl.de/#/app/home GDSC [108] v.8.2: https://www.cancerrxgene.org/ GnomAD [54] v.2.1.1: https://gnomad.broadinstitute.org/ GTEx [92] v.8: https://gtexportal.org/home/ HPRD [96] v.9: https://www.hprd.org/ IntAct [94] v.4.2.14: https://www.ebi.ac.uk/intact/home KEGG [106] v.94.1: https://www.genome.jp/kegg/ Meta-KB [109] v.1: https://cancervariants.org/ MiRecords [101] v.8.0: http://c1.accurascience.com/miRecords/ MiTarBase [100] v.4.0: https://mirtarbase.cuhk.edu.cn/~miRTarBase/miRTarBase_2022/php/index.php NCI Genomics Data Commons Portal [73]: https://gdc.cancer.gov/ PICKLES [61]: https://pickles.hart-lab.org/ Protein Atlas [91] v.19.3: https://www.proteinatlas.org/ Reactome [99] v.72: https://reactome.org/ RefSeq [87] v.99: https://www.ncbi.nlm.nih.gov/refseq/

## References

[1] Network CGAR (2008). Comprehensive genomic characterization defines human glioblastoma genes and core pathways. Nature..

[2] Hudson TJ, Anderson W, Artez A, Barker AD, Bell C, International Cancer Genome C (2010). International network of cancer genome projects. Nature.

[3] Hutter C, Zenklusen JC (2018). The Cancer Genome Atlas: creating lasting value beyond its data. Cell..

[4] Pon JR, Marra MA (2015). Driver and passenger mutations in cancer. Annu Rev Pathol.

[5] Porta-Pardo E, Kamburov A, Tamborero D, Pons T, Grases D, Valencia A, Lopez-Bigas N, Getz G, Godzik A (2017). Comparison of algorithms for the detection of cancer drivers at subgene resolution. Nat Methods.

[6] Martínez-Jiménez F, Muiños F, Sentís I, Deu-Pons J, Reyes-Salazar I, Arnedo-Pac C, Mularoni L, Pich O, Bonet J, Kranas H, Gonzalez-Perez A, Lopez-Bigas N (2020). A compendium of mutational cancer driver genes. Nat Rev Cancer.

[7] Bailey MH, Tokheim C, Porta-Pardo E, Sengupta S, Bertrand D, Weerasinghe A, Colaprico A, Wendl MC, Kim J, Reardon B, Ng PKS, Jeong KJ, Cao S, Wang Z, Gao J, Gao Q, Wang F, Liu EM, Mularoni L, Rubio-Perez C, Nagarajan N, Cortés-Ciriano I, Zhou DC, Liang WW, Hess JM, Yellapantula VD, Tamborero D, Gonzalez-Perez A, Suphavilai C, Ko JY, Khurana E, Park PJ, van Allen EM, Liang H, Lawrence MS, Godzik A, Lopez-Bigas N, Stuart J, Wheeler D, Getz G, Chen K, Lazar AJ, Mills GB, Karchin R, Ding L, Caesar-Johnson SJ, Demchok JA, Felau I, Kasapi M, Ferguson ML, Hutter CM, Sofia HJ, Tarnuzzer R, Wang Z, Yang L, Zenklusen JC, Zhang J(J), Chudamani S, Liu J, Lolla L, Naresh R, Pihl T, Sun Q, Wan Y, Wu Y, Cho J, DeFreitas T, Frazer S, Gehlenborg N, Getz G, Heiman DI, Kim J, Lawrence MS, Lin P, Meier S, Noble MS, Saksena G, Voet D, Zhang H, Bernard B, Chambwe N, Dhankani V, Knijnenburg T, Kramer R, Leinonen K, Liu Y, Miller M, Reynolds S, Shmulevich I, Thorsson V, Zhang W, Akbani R, Broom BM, Hegde AM, Ju Z, Kanchi RS, Korkut A, Li J, Liang H, Ling S, Liu W, Lu Y, Mills GB, Ng KS, Rao A, Ryan M, Wang J, Weinstein JN, Zhang J, Abeshouse A, Armenia J, Chakravarty D, Chatila WK, de Bruijn I, Gao J, Gross BE, Heins ZJ, Kundra R, la K, Ladanyi M, Luna A, Nissan MG, Ochoa A, Phillips SM, Reznik E, Sanchez-Vega F, Sander C, Schultz N, Sheridan R, Sumer SO, Sun Y, Taylor BS, Wang J, Zhang H, Anur P, Peto M, Spellman P, Benz C, Stuart JM, Wong CK, Yau C, Hayes DN, Parker JS, Wilkerson MD, Ally A, Balasundaram M, Bowlby R, Brooks D, Carlsen R, Chuah E, Dhalla N, Holt R, Jones SJM, Kasaian K, Lee D, Ma Y, Marra MA, Mayo M, Moore RA, Mungall AJ, Mungall K, Robertson AG, Sadeghi S, Schein JE, Sipahimalani P, Tam A, Thiessen N, Tse K, Wong T, Berger AC, Beroukhim R, Cherniack AD, Cibulskis C, Gabriel SB, Gao GF, Ha G, Meyerson M, Schumacher SE, Shih J, Kucherlapati MH, Kucherlapati RS, Baylin S, Cope L, Danilova L, Bootwalla MS, Lai PH, Maglinte DT, van den Berg DJ, Weisenberger DJ, Auman JT, Balu S, Bodenheimer T, Fan C, Hoadley KA, Hoyle AP, Jefferys SR, Jones CD, Meng S, Mieczkowski PA, Mose LE, Perou AH, Perou CM, Roach J, Shi Y, Simons JV, Skelly T, Soloway MG, Tan D, Veluvolu U, Fan H, Hinoue T, Laird PW, Shen H, Zhou W, Bellair M, Chang K, Covington K, Creighton CJ, Dinh H, Doddapaneni HV, Donehower LA, Drummond J, Gibbs RA, Glenn R, Hale W, Han Y, Hu J, Korchina V, Lee S, Lewis L, Li W, Liu X, Morgan M, Morton D, Muzny D, Santibanez J, Sheth M, Shinbrot E, Wang L, Wang M, Wheeler DA, Xi L, Zhao F, Hess J, Appelbaum EL, Bailey M, Cordes MG, Ding L, Fronick CC, Fulton LA, Fulton RS, Kandoth C, Mardis ER, McLellan MD, Miller CA, Schmidt HK, Wilson RK, Crain D, Curley E, Gardner J, Lau K, Mallery D, Morris S, Paulauskis J, Penny R, Shelton C, Shelton T, Sherman M, Thompson E, Yena P, Bowen J, Gastier-Foster JM, Gerken M, Leraas KM, Lichtenberg TM, Ramirez NC, Wise L, Zmuda E, Corcoran N, Costello T, Hovens C, Carvalho AL, de Carvalho AC, Fregnani JH, Longatto-Filho A, Reis RM, Scapulatempo-Neto C, Silveira HCS, Vidal DO, Burnette A, Eschbacher J, Hermes B, Noss A, Singh R, Anderson ML, Castro PD, Ittmann M, Huntsman D, Kohl B, le X, Thorp R, Andry C, Duffy ER, Lyadov V, Paklina O, Setdikova G, Shabunin A, Tavobilov M, McPherson C, Warnick R, Berkowitz R, Cramer D, Feltmate C, Horowitz N, Kibel A, Muto M, Raut CP, Malykh A, Barnholtz-Sloan JS, Barrett W, Devine K, Fulop J, Ostrom QT, Shimmel K, Wolinsky Y, Sloan AE, de Rose A, Giuliante F, Goodman M, Karlan BY, Hagedorn CH, Eckman J, Harr J, Myers J, Tucker K, Zach LA, Deyarmin B, Hu H, Kvecher L, Larson C, Mural RJ, Somiari S, Vicha A, Zelinka T, Bennett J, Iacocca M, Rabeno B, Swanson P, Latour M, Lacombe L, Têtu B, Bergeron A, McGraw M, Staugaitis SM, Chabot J, Hibshoosh H, Sepulveda A, Su T, Wang T, Potapova O, Voronina O, Desjardins L, Mariani O, Roman-Roman S, Sastre X, Stern MH, Cheng F, Signoretti S, Berchuck A, Bigner D, Lipp E, Marks J, McCall S, McLendon R, Secord A, Sharp A, Behera M, Brat DJ, Chen A, Delman K, Force S, Khuri F, Magliocca K, Maithel S, Olson JJ, Owonikoko T, Pickens A, Ramalingam S, Shin DM, Sica G, van Meir EG, Zhang H, Eijckenboom W, Gillis A, Korpershoek E, Looijenga L, Oosterhuis W, Stoop H, van Kessel KE, Zwarthoff EC, Calatozzolo C, Cuppini L, Cuzzubbo S, DiMeco F, Finocchiaro G, Mattei L, Perin A, Pollo B, Chen C, Houck J, Lohavanichbutr P, Hartmann A, Stoehr C, Stoehr R, Taubert H, Wach S, Wullich B, Kycler W, Murawa D, Wiznerowicz M, Chung K, Edenfield WJ, Martin J, Baudin E, Bubley G, Bueno R, de Rienzo A, Richards WG, Kalkanis S, Mikkelsen T, Noushmehr H, Scarpace L, Girard N, Aymerich M, Campo E, Giné E, Guillermo AL, van Bang N, Hanh PT, Phu BD, Tang Y, Colman H, Evason K, Dottino PR, Martignetti JA, Gabra H, Juhl H, Akeredolu T, Stepa S, Hoon D, Ahn K, Kang KJ, Beuschlein F, Breggia A, Birrer M, Bell D, Borad M, Bryce AH, Castle E, Chandan V, Cheville J, Copland JA, Farnell M, Flotte T, Giama N, Ho T, Kendrick M, Kocher JP, Kopp K, Moser C, Nagorney D, O’Brien D, O’Neill BP, Patel T, Petersen G, Que F, Rivera M, Roberts L, Smallridge R, Smyrk T, Stanton M, Thompson RH, Torbenson M, Yang JD, Zhang L, Brimo F, Ajani JA, Gonzalez AMA, Behrens C, Bondaruk J, Broaddus R, Czerniak B, Esmaeli B, Fujimoto J, Gershenwald J, Guo C, Lazar AJ, Logothetis C, Meric-Bernstam F, Moran C, Ramondetta L, Rice D, Sood A, Tamboli P, Thompson T, Troncoso P, Tsao A, Wistuba I, Carter C, Haydu L, Hersey P, Jakrot V, Kakavand H, Kefford R, Lee K, Long G, Mann G, Quinn M, Saw R, Scolyer R, Shannon K, Spillane A, Stretch J, Synott M, Thompson J, Wilmott J, al-Ahmadie H, Chan TA, Ghossein R, Gopalan A, Levine DA, Reuter V, Singer S, Singh B, Tien NV, Broudy T, Mirsaidi C, Nair P, Drwiega P, Miller J, Smith J, Zaren H, Park JW, Hung NP, Kebebew E, Linehan WM, Metwalli AR, Pacak K, Pinto PA, Schiffman M, Schmidt LS, Vocke CD, Wentzensen N, Worrell R, Yang H, Moncrieff M, Goparaju C, Melamed J, Pass H, Botnariuc N, Caraman I, Cernat M, Chemencedji I, Clipca A, Doruc S, Gorincioi G, Mura S, Pirtac M, Stancul I, Tcaciuc D, Albert M, Alexopoulou I, Arnaout A, Bartlett J, Engel J, Gilbert S, Parfitt J, Sekhon H, Thomas G, Rassl DM, Rintoul RC, Bifulco C, Tamakawa R, Urba W, Hayward N, Timmers H, Antenucci A, Facciolo F, Grazi G, Marino M, Merola R, de Krijger R, Gimenez-Roqueplo AP, Piché A, Chevalier S, McKercher G, Birsoy K, Barnett G, Brewer C, Farver C, Naska T, Pennell NA, Raymond D, Schilero C, Smolenski K, Williams F, Morrison C, Borgia JA, Liptay MJ, Pool M, Seder CW, Junker K, Omberg L, Dinkin M, Manikhas G, Alvaro D, Bragazzi MC, Cardinale V, Carpino G, Gaudio E, Chesla D, Cottingham S, Dubina M, Moiseenko F, Dhanasekaran R, Becker KF, Janssen KP, Slotta-Huspenina J, Abdel-Rahman MH, Aziz D, Bell S, Cebulla CM, Davis A, Duell R, Elder JB, Hilty J, Kumar B, Lang J, Lehman NL, Mandt R, Nguyen P, Pilarski R, Rai K, Schoenfield L, Senecal K, Wakely P, Hansen P, Lechan R, Powers J, Tischler A, Grizzle WE, Sexton KC, Kastl A, Henderson J, Porten S, Waldmann J, Fassnacht M, Asa SL, Schadendorf D, Couce M, Graefen M, Huland H, Sauter G, Schlomm T, Simon R, Tennstedt P, Olabode O, Nelson M, Bathe O, Carroll PR, Chan JM, Disaia P, Glenn P, Kelley RK, Landen CN, Phillips J, Prados M, Simko J, Smith-McCune K, VandenBerg S, Roggin K, Fehrenbach A, Kendler A, Sifri S, Steele R, Jimeno A, Carey F, Forgie I, Mannelli M, Carney M, Hernandez B, Campos B, Herold-Mende C, Jungk C, Unterberg A, von Deimling A, Bossler A, Galbraith J, Jacobus L, Knudson M, Knutson T, Ma D, Milhem M, Sigmund R, Godwin AK, Madan R, Rosenthal HG, Adebamowo C, Adebamowo SN, Boussioutas A, Beer D, Giordano T, Mes-Masson AM, Saad F, Bocklage T, Landrum L, Mannel R, Moore K, Moxley K, Postier R, Walker J, Zuna R, Feldman M, Valdivieso F, Dhir R, Luketich J, Pinero EMM, Quintero-Aguilo M, Carlotti CG, Dos Santos JS, Kemp R, Sankarankuty A, Tirapelli D, Catto J, Agnew K, Swisher E, Creaney J, Robinson B, Shelley CS, Godwin EM, Kendall S, Shipman C, Bradford C, Carey T, Haddad A, Moyer J, Peterson L, Prince M, Rozek L, Wolf G, Bowman R, Fong KM, Yang I, Korst R, Rathmell WK, Fantacone-Campbell JL, Hooke JA, Kovatich AJ, Shriver CD, DiPersio J, Drake B, Govindan R, Heath S, Ley T, van Tine B, Westervelt P, Rubin MA, Lee JI, Aredes ND, Mariamidze A (2018). Comprehensive characterization of cancer driver genes and mutations. Cell..

[8] Consortium ITP-CAoWG (2020). Pan-cancer analysis of whole genomes. Nature..

[9] Kandoth C, McLellan MD, Vandin F, Ye K, Niu B, Lu C (2013). Mutational landscape and significance across 12 major cancer types. Nature..

[10] Wijewardhane N, Dressler L, Ciccarelli FD (2021). Normal somatic mutations in cancer transformation. Cancer Cell.

[11] Kakiuchi N, Ogawa S (2021). Clonal expansion in non-cancer tissues. Nat Rev Cancer.

[12] Martincorena I, Roshan A, Gerstung M, Ellis P, Van Loo P, McLaren S (2015). Tumor evolution. High burden and pervasive positive selection of somatic mutations in normal human skin. Science..

[13] Tang J, Fewings E, Chang D, Zeng H, Liu S, Jorapur A, Belote RL, McNeal AS, Tan TM, Yeh I, Arron ST, Judson-Torres RL, Bastian BC, Shain AH (2020). The genomic landscapes of individual melanocytes from human skin. Nature..

[14] Yokoyama A, Kakiuchi N, Yoshizato T, Nannya Y, Suzuki H, Takeuchi Y, Shiozawa Y, Sato Y, Aoki K, Kim SK, Fujii Y, Yoshida K, Kataoka K, Nakagawa MM, Inoue Y, Hirano T, Shiraishi Y, Chiba K, Tanaka H, Sanada M, Nishikawa Y, Amanuma Y, Ohashi S, Aoyama I, Horimatsu T, Miyamoto S’, Tsunoda S, Sakai Y, Narahara M, Brown JB, Sato Y, Sawada G, Mimori K, Minamiguchi S, Haga H, Seno H, Miyano S, Makishima H, Muto M, Ogawa S (2019). Age-related remodelling of oesophageal epithelia by mutated cancer drivers. Nature..

[15] Martincorena I, Fowler JC, Wabik A, Lawson ARJ, Abascal F, Hall MWJ, Cagan A, Murai K, Mahbubani K, Stratton MR, Fitzgerald RC, Handford PA, Campbell PJ, Saeb-Parsy K, Jones PH (2018). Somatic mutant clones colonize the human esophagus with age. Science..

[16] Suda K, Nakaoka H, Yoshihara K, Ishiguro T, Tamura R, Mori Y, Yamawaki K, Adachi S, Takahashi T, Kase H, Tanaka K, Yamamoto T, Motoyama T, Inoue I, Enomoto T (2018). Clonal expansion and diversification of cancer-associated mutations in endometriosis and normal endometrium. Cell Rep.

[17] Vogelstein B, Papadopoulos N, Velculescu VE, Zhou S, Diaz LA, Kinzler KW (2013). Cancer genome landscapes. Science..

[18] Saito Y, Koya J, Araki M, Kogure Y, Shingaki S, Tabata M, McClure MB, Yoshifuji K, Matsumoto S, Isaka Y, Tanaka H, Kanai T, Miyano S, Shiraishi Y, Okuno Y, Kataoka K (2020). Landscape and function of multiple mutations within individual oncogenes. Nature..

[19] Sondka Z, Bamford S, Cole CG, Ward SA, Dunham I, Forbes SA (2018). The COSMIC Cancer Gene Census: describing genetic dysfunction across all human cancers. Nat Rev Cancer.

[20] Liu EM, Martinez-Fundichely A, Bollapragada R, Spiewack M, Khurana E (2021). CNCDatabase: a database of non-coding cancer drivers. Nucleic Acids Res.

[21] Campbell PJ, Getz G, Korbel JO, Stuart JM, Jennings JL, Stein LD (2020). Pan-cancer analysis of whole genomes. Nature..

[22] Hornshoj H, Nielsen MM, Sinnott-Armstrong NA, Switnicki MP, Juul M, Madsen T (2018). Pan-cancer screen for mutations in non-coding elements with conservation and cancer specificity reveals correlations with expression and survival. NPJ Genom Med.

[23] Juul M, Bertl J, Guo Q, Nielsen MM, Switnicki M, Hornshoj H, et al. Non-coding cancer driver candidates identified with a sample- and position-specific model of the somatic mutation rate. Elife. 2017;6. 10.7554/eLife.21778. 10.7554/eLife.21778 PMC5440169 28362259

[24] Zhu H, Uuskula-Reimand L, Isaev K, Wadi L, Alizada A, Shuai S (2020). Candidate cancer driver mutations in distal regulatory elements and long-range chromatin interaction networks. Mol Cell.

[25] Lanzos A, Carlevaro-Fita J, Mularoni L, Reverter F, Palumbo E, Guigo R (2017). Discovery of cancer driver long noncoding RNAs across 1112 tumour genomes: new candidates and distinguishing features. Sci Rep.

[26] Mularoni L, Sabarinathan R, Deu-Pons J, Gonzalez-Perez A, Lopez-Bigas N (2016). OncodriveFML: a general framework to identify coding and non-coding regions with cancer driver mutations. Genome Biol.

[27] Cornish AJ, Hoang PH, Dobbins SE, Law PJ, Chubb D, Orlando G, Houlston RS (2019). Identification of recurrent noncoding mutations in B-cell lymphoma using capture Hi-C. Blood Adv.

[28] Hayward NK, Wilmott JS, Waddell N, Johansson PA, Field MA, Nones K, Patch AM, Kakavand H, Alexandrov LB, Burke H, Jakrot V, Kazakoff S, Holmes O, Leonard C, Sabarinathan R, Mularoni L, Wood S, Xu Q, Waddell N, Tembe V, Pupo GM, de Paoli-Iseppi R, Vilain RE, Shang P, Lau LMS, Dagg RA, Schramm SJ, Pritchard A, Dutton-Regester K, Newell F, Fitzgerald A, Shang CA, Grimmond SM, Pickett HA, Yang JY, Stretch JR, Behren A, Kefford RF, Hersey P, Long GV, Cebon J, Shackleton M, Spillane AJ, Saw RPM, López-Bigas N, Pearson JV, Thompson JF, Scolyer RA, Mann GJ (2017). Whole-genome landscapes of major melanoma subtypes. Nature..

[29] Botlagunta M, Vesuna F, Mironchik Y, Raman A, Lisok A, Winnard P, Mukadam S, van Diest P, Chen JH, Farabaugh P, Patel AH, Raman V (2008). Oncogenic role of DDX3 in breast cancer biogenesis. Oncogene..

[30] Pu J, Wang J, Qin Z, Wang A, Zhang Y, Wu X, Wu Y, Li W, Xu Z, Lu Y, Tang Q, Wei H (2020). IGF2BP2 promotes liver cancer growth through an m6A-FEN1-dependent mechanism. Front Oncol.

[31] Sun X, Jia M, Sun W, Feng L, Gu C, Wu T (2019). Functional role of RBM10 in lung adenocarcinoma proliferation. Int J Oncol.

[32] Soussi T, Wiman KG (2015). TP53: an oncogene in disguise. Cell Death Differ.

[33] Yang MH, Chang SY, Chiou SH, Liu CJ, Chi CW, Chen PM, Teng SC, Wu KJ (2007). Overexpression of NBS1 induces epithelial-mesenchymal transition and co-expression of NBS1 and Snail predicts metastasis of head and neck cancer. Oncogene..

[34] Manandhar S, Kim CG, Lee SH, Kang SH, Basnet N, Lee YM (2017). Exostosin 1 regulates cancer cell stemness in doxorubicin-resistant breast cancer cells. Oncotarget..

[35] Li A, Zhu X, Wang C, Yang S, Qiao Y, Qiao R, Zhang J (2019). Upregulation of NDRG1 predicts poor outcome and facilitates disease progression by influencing the EMT process in bladder cancer. Sci Rep.

[36] Meacham CE, Lawton LN, Soto-Feliciano YM, Pritchard JR, Joughin BA, Ehrenberger T, Fenouille N, Zuber J, Williams RT, Young RA, Hemann MT (2015). A genome-scale in vivo loss-of-function screen identifies Phf6 as a lineage-specific regulator of leukemia cell growth. Genes Dev.

[37] Sesen J, Casaos J, Scotland SJ, Seva C, Eisinger-Mathason TS, Skuli N (2017). The bad, the good and eIF3e/INT6. Front Biosci (Landmark Ed).

[38] Shi J, Zhang L, Zhou D, Zhang J, Lin Q, Guan W, Zhang J, Ren W, Xu G (2018). Biological function of ribosomal protein L10 on cell behavior in human epithelial ovarian cancer. J Cancer.

[39] Liu P, Morrison C, Wang L, Xiong D, Vedell P, Cui P, Hua X, Ding F, Lu Y, James M, Ebben JD, Xu H, Adjei AA, Head K, Andrae JW, Tschannen MR, Jacob H, Pan J, Zhang Q, van den Bergh F, Xiao H, Lo KC, Patel J, Richmond T, Watt MA, Albert T, Selzer R, Anderson M, Wang J, Wang Y, Starnes S, Yang P, You M (2012). Identification of somatic mutations in non-small cell lung carcinomas using whole-exome sequencing. Carcinogenesis..

[40] Lai MW, Liang KH, Lin WR, Huang YH, Huang SF, Chen TC, Yeh CT (2016). Hepatocarcinogenesis in transgenic mice carrying hepatitis B virus pre-S/S gene with the sW172* mutation. Oncogenesis..

[41] Cai C, Cooper GF, Lu KN, Ma X, Xu S, Zhao Z, Chen X, Xue Y, Lee AV, Clark N, Chen V, Lu S, Chen L, Yu L, Hochheiser HS, Jiang X, Wang QJ, Lu X (2019). Systematic discovery of the functional impact of somatic genome alterations in individual tumors through tumor-specific causal inference. PLoS Comput Biol.

[42] Lawrence MS, Stojanov P, Polak P, Kryukov GV, Cibulskis K, Sivachenko A, Carter SL, Stewart C, Mermel CH, Roberts SA, Kiezun A, Hammerman PS, McKenna A, Drier Y, Zou L, Ramos AH, Pugh TJ, Stransky N, Helman E, Kim J, Sougnez C, Ambrogio L, Nickerson E, Shefler E, Cortés ML, Auclair D, Saksena G, Voet D, Noble M, DiCara D, Lin P, Lichtenstein L, Heiman DI, Fennell T, Imielinski M, Hernandez B, Hodis E, Baca S, Dulak AM, Lohr J, Landau DA, Wu CJ, Melendez-Zajgla J, Hidalgo-Miranda A, Koren A, McCarroll SA, Mora J, Lee RS, Crompton B, Onofrio R, Parkin M, Winckler W, Ardlie K, Gabriel SB, Roberts CWM, Biegel JA, Stegmaier K, Bass AJ, Garraway LA, Meyerson M, Golub TR, Gordenin DA, Sunyaev S, Lander ES, Getz G (2013). Mutational heterogeneity in cancer and the search for new cancer-associated genes. Nature..

[43] Hannon GJ, Beach D (1994). pl5INK4B is a potentia| effector of TGF-β-induced cell cycle arrest. Nature.

[44] Syed AS, D’Antonio M, Ciccarelli FD (2010). Network of Cancer Genes: a web resource to analyze duplicability, orthology and network properties of cancer genes. Nucleic Acids Res.

[45] Trigos AS, Pearson RB, Papenfuss AT, Goode DL (2019). Somatic mutations in early metazoan genes disrupt regulatory links between unicellular and multicellular genes in cancer. eLife..

[46] Rambaldi D, Giorgi FM, Capuani F, Ciliberto A, Ciccarelli FD (2008). Low duplicability and network fragility of cancer genes. Trends Genet.

[47] Domazet-Loso T, Tautz D (2010). Phylostratigraphic tracking of cancer genes suggests a link to the emergence of multicellularity in metazoa. BMC Biol.

[48] D’Antonio M, Ciccarelli FD (2013). Integrated analysis of recurrent properties of cancer genes to identify novel drivers. Genome Biol.

[49] Ostrow SL, Barshir R, DeGregori J, Yeger-Lotem E, Hershberg R (2014). Cancer evolution is associated with pervasive positive selection on globally expressed genes. PLoS Genet.

[50] An O, Dall’Olio GM, Mourikis TP, Ciccarelli FD (2016). NCG 5.0: updates of a manually curated repository of cancer genes and associated properties from cancer mutational screenings. Nucleic Acids Res.

[51] Jonsson PF, Bates PA (2006). Global topological features of cancer proteins in the human interactome. Bioinformatics..

[52] Xia J, Sun J, Jia P, Zhao Z (2011). Do cancer proteins really interact strongly in the human protein-protein interaction network?. Comput Biol Chem.

[53] D’Antonio M, Ciccarelli FD (2011). Modification of gene duplicability during the evolution of protein interaction network. PLoS Comput Biol.

[54] Karczewski KJ, Francioli LC, Tiao G, Cummings BB, Alföldi J, Wang Q, Collins RL, Laricchia KM, Ganna A, Birnbaum DP, Gauthier LD, Brand H, Solomonson M, Watts NA, Rhodes D, Singer-Berk M, England EM, Seaby EG, Kosmicki JA, Walters RK, Tashman K, Farjoun Y, Banks E, Poterba T, Wang A, Seed C, Whiffin N, Chong JX, Samocha KE, Pierce-Hoffman E, Zappala Z, O’Donnell-Luria AH, Minikel EV, Weisburd B, Lek M, Ware JS, Vittal C, Armean IM, Bergelson L, Cibulskis K, Connolly KM, Covarrubias M, Donnelly S, Ferriera S, Gabriel S, Gentry J, Gupta N, Jeandet T, Kaplan D, Llanwarne C, Munshi R, Novod S, Petrillo N, Roazen D, Ruano-Rubio V, Saltzman A, Schleicher M, Soto J, Tibbetts K, Tolonen C, Wade G, Talkowski ME, Aguilar Salinas CA, Ahmad T, Albert CM, Ardissino D, Atzmon G, Barnard J, Beaugerie L, Benjamin EJ, Boehnke M, Bonnycastle LL, Bottinger EP, Bowden DW, Bown MJ, Chambers JC, Chan JC, Chasman D, Cho J, Chung MK, Cohen B, Correa A, Dabelea D, Daly MJ, Darbar D, Duggirala R, Dupuis J, Ellinor PT, Elosua R, Erdmann J, Esko T, Färkkilä M, Florez J, Franke A, Getz G, Glaser B, Glatt SJ, Goldstein D, Gonzalez C, Groop L, Haiman C, Hanis C, Harms M, Hiltunen M, Holi MM, Hultman CM, Kallela M, Kaprio J, Kathiresan S, Kim BJ, Kim YJ, Kirov G, Kooner J, Koskinen S, Krumholz HM, Kugathasan S, Kwak SH, Laakso M, Lehtimäki T, Loos RJF, Lubitz SA, Ma RCW, MacArthur DG, Marrugat J, Mattila KM, McCarroll S, McCarthy MI, McGovern D, McPherson R, Meigs JB, Melander O, Metspalu A, Neale BM, Nilsson PM, O’Donovan MC, Ongur D, Orozco L, Owen MJ, Palmer CNA, Palotie A, Park KS, Pato C, Pulver AE, Rahman N, Remes AM, Rioux JD, Ripatti S, Roden DM, Saleheen D, Salomaa V, Samani NJ, Scharf J, Schunkert H, Shoemaker MB, Sklar P, Soininen H, Sokol H, Spector T, Sullivan PF, Suvisaari J, Tai ES, Teo YY, Tiinamaija T, Tsuang M, Turner D, Tusie-Luna T, Vartiainen E, Vawter MP, Ware JS, Watkins H, Weersma RK, Wessman M, Wilson JG, Xavier RJ, Neale BM, Daly MJ, MacArthur DG, Genome Aggregation Database Consortium (2020). The mutational constraint spectrum quantified from variation in 141,456 humans. Nature..

[55] Dempster JM, Rossen J, Kazachkova M, Pan J, Kugener G, Root DE, et al. Extracting biological insights from the project Achilles genome-Scale CRISPR screens in cancer cell lines. BioRxiv. 2019;720243. 10.1101/720243.

[56] Broad D. DepMap 20Q3 Public, figshare. Dataset. 2020. 10.6084/m9.figshare.12931238.v1.

[57] Meyers RM, Bryan JG, McFarland JM, Weir BA, Sizemore AE, Xu H (2017). Computational correction of copy number effect improves specificity of CRISPR-Cas9 essentiality screens in cancer cells. Nat Genet.

[58] Behan FM, Iorio F, Picco G, Goncalves E, Beaver CM, Migliardi G (2019). Prioritization of cancer therapeutic targets using CRISPR-Cas9 screens. Nature..

[59] DepMap Broad. Project SCORE processed with CERES. figshare. Dataset. 2019. 10.6084/m9.figshare.9116732.

[60] DepMap Broad. DepMap GeCKO 19Q1. figshare. Fileset. 2019. 10.6084/m9.figshare.7668407.

[61] Lenoir WF, Lim TL, Hart T (2018). PICKLES: the database of pooled in-vitro CRISPR knockout library essentiality screens. Nucleic Acids Res.

[62] McFarland JM, Ho ZV, Kugener G, Dempster JM, Montgomery PG, Bryan JG (2018). Improved estimation of cancer dependencies from large-scale RNAi screens using model-based normalization and data integration. Nat Commun.

[63] Tsherniak A, Vazquez F, Montgomery PG, Weir BA, Kryukov G, Cowley GS, Gill S, Harrington WF, Pantel S, Krill-Burger JM, Meyers RM, Ali L, Goodale A, Lee Y, Jiang G, Hsiao J, Gerath WFJ, Howell S, Merkel E, Ghandi M, Garraway LA, Root DE, Golub TR, Boehm JS, Hahn WC (2017). Defining a cancer dependency map. Cell..

[64] Bertrand D, Chng KR, Sherbaf FG, Kiesel A, Chia BK, Sia YY (2015). Patient-specific driver gene prediction and risk assessment through integrated network analysis of cancer omics profiles. Nucleic Acids Res.

[65] Bashashati A, Haffari G, Ding J, Ha G, Lui K, Rosner J, Huntsman DG, Caldas C, Aparicio SA, Shah SP (2012). DriverNet: uncovering the impact of somatic driver mutations on transcriptional networks in cancer. Genome Biol.

[66] Hou JP, Ma J (2014). DawnRank: discovering personalized driver genes in cancer. Genome Med.

[67] Dong C, Guo Y, Yang H, He Z, Liu X, Wang K (2016). iCAGES: integrated CAncer GEnome Score for comprehensively prioritizing driver genes in personal cancer genomes. Genome Med.

[68] Nulsen J, Misetic H, Yau C, Ciccarelli FD (2021). Pan-cancer detection of driver genes at the single-patient resolution. Genome Med.

[69] Mourikis TP, Benedetti L, Foxall E, Temelkovski D, Nulsen J, Perner J (2019). Patient-specific cancer genes contribute to recurrently perturbed pathways and establish therapeutic vulnerabilities in esophageal adenocarcinoma. Nat Commun.

[70] Repana D, Nulsen J, Dressler L, Bortolomeazzi M, Venkata SK, Tourna A, Yakovleva A, Palmieri T, Ciccarelli FD (2019). The Network of Cancer Genes (NCG): a comprehensive catalogue of known and candidate cancer genes from cancer sequencing screens. Genome Biol.

[71] Tate JG, Bamford S, Jubb HC, Sondka Z, Beare DM, Bindal N, Boutselakis H, Cole CG, Creatore C, Dawson E, Fish P, Harsha B, Hathaway C, Jupe SC, Kok CY, Noble K, Ponting L, Ramshaw CC, Rye CE, Speedy HE, Stefancsik R, Thompson SL, Wang S, Ward S, Campbell PJ, Forbes SA (2019). COSMIC: the Catalogue Of Somatic Mutations In Cancer. Nucleic Acids Res.

[72] Hoadley KA, Yau C, Hinoue T, Wolf DM, Lazar AJ, Drill E (2018). Cell-of-origin patterns dominate the molecular classification of 10,000 tumors from 33 types of cancer. Cell.

[73] Grossman RL, Heath AP, Ferretti V, Varmus HE, Lowy DR, Kibbe WA, Staudt LM (2016). Toward a shared vision for cancer genomic data. N Engl J Med.

[74] Wang K, Li M, Hakonarson H (2010). ANNOVAR: functional annotation of genetic variants from high-throughput sequencing data. Nucleic Acids Res.

[75] Liu X, Wu C, Li C, Boerwinkle E (2016). dbNSFP v3.0: a one-stop database of functional predictions and annotations for human nonsynonymous and splice-site SNVs. Hum Mutat.

[76] Ng PC, Henikoff S (2003). SIFT: predicting amino acid changes that affect protein function. Nucleic Acids Res.

[77] Adzhubei I, Jordan DM, Sunyaev SR (2013). Predicting functional effect of human missense mutations using PolyPhen-2. Curr Protoc Hum Genet.

[78] Schwarz JM, Rödelsperger C, Schuelke M, Seelow D (2010). MutationTaster evaluates disease-causing potential of sequence alterations. Nat Methods.

[79] Reva B, Antipin Y, Sander C (2011). Predicting the functional impact of protein mutations: application to cancer genomics. Nucleic Acids Res.

[80] Chun S, Fay JC (2009). Identification of deleterious mutations within three human genomes. Genome Res.

[81] Shihab HA, Gough J, Cooper DN, Stenson PD, Barker GL, Edwards KJ (2013). Predicting the functional, molecular, and phenotypic consequences of amino acid substitutions using hidden Markov models. Hum Mutat.

[82] Pollard KS, Hubisz MJ, Rosenbloom KR, Siepel A (2010). Detection of nonneutral substitution rates on mammalian phylogenies. Genome Res.

[83] Davydov EV, Goode DL, Sirota M, Cooper GM, Sidow A, Batzoglou S (2010). Identifying a high fraction of the human genome to be under selective constraint using GERP++. PLoS Comput Biol.

[84] Garber M, Guttman M, Clamp M, Zody MC, Friedman N, Xie X (2009). Identifying novel constrained elements by exploiting biased substitution patterns. Bioinformatics..

[85] Tamborero D, Gonzalez-Perez A, Lopez-Bigas N (2013). OncodriveCLUST: exploiting the positional clustering of somatic mutations to identify cancer genes. Bioinformatics..

[86] Van Loo P, Nordgard SH, Lingjærde OC, Russnes HG, Rye IH, Sun W (2010). Allele-specific copy number analysis of tumors. Proc Natl Acad Sci U S A.

[87] O’Leary NA, Wright MW, Brister JR, Ciufo S, Haddad D, McVeigh R (2016). Reference sequence (RefSeq) database at NCBI: current status, taxonomic expansion, and functional annotation. Nucleic Acids Res.

[88] Kent WJ (2002). BLAT—the BLAST-like alignment tool. Genome Res.

[89] Bhagwat M, Young L, Robison RR (2012). Using BLAT to find sequence similarity in closely related genomes. Curr Protoc Bioinformatics.

[90] Huerta-Cepas J, Szklarczyk D, Heller D, Hernández-Plaza A, Forslund SK, Cook H, Mende DR, Letunic I, Rattei T, Jensen LJ, von Mering C, Bork P (2019). eggNOG 5.0: a hierarchical, functionally and phylogenetically annotated orthology resource based on 5090 organisms and 2502 viruses. Nucleic Acids Res.

[91] Uhlén M, Fagerberg L, Hallström BM, Lindskog C, Oksvold P, Mardinoglu A, Sivertsson Å, Kampf C, Sjöstedt E, Asplund A, Olsson IM, Edlund K, Lundberg E, Navani S, Szigyarto CAK, Odeberg J, Djureinovic D, Takanen JO, Hober S, Alm T, Edqvist PH, Berling H, Tegel H, Mulder J, Rockberg J, Nilsson P, Schwenk JM, Hamsten M, von Feilitzen K, Forsberg M, Persson L, Johansson F, Zwahlen M, von Heijne G, Nielsen J, Pontén F (2015). Proteomics. Tissue-based map of the human proteome. Science.

[92] Consortium G (2020). The GTEx Consortium atlas of genetic regulatory effects across human tissues. Science..

[93] Oughtred R, Stark C, Breitkreutz BJ, Rust J, Boucher L, Chang C, Kolas N, O’Donnell L, Leung G, McAdam R, Zhang F, Dolma S, Willems A, Coulombe-Huntington J, Chatr-aryamontri A, Dolinski K, Tyers M (2019). The BioGRID interaction database: 2019 update. Nucleic Acids Res.

[94] Orchard S, Ammari M, Aranda B, Breuza L, Briganti L, Broackes-Carter F, Campbell NH, Chavali G, Chen C, del-Toro N, Duesbury M, Dumousseau M, Galeota E, Hinz U, Iannuccelli M, Jagannathan S, Jimenez R, Khadake J, Lagreid A, Licata L, Lovering RC, Meldal B, Melidoni AN, Milagros M, Peluso D, Perfetto L, Porras P, Raghunath A, Ricard-Blum S, Roechert B, Stutz A, Tognolli M, van Roey K, Cesareni G, Hermjakob H (2014). The MIntAct project--IntAct as a common curation platform for 11 molecular interaction databases. Nucleic Acids Res.

[95] Salwinski L, Miller CS, Smith AJ, Pettit FK, Bowie JU, Eisenberg D (2004). The database of interacting proteins: 2004 update. Nucleic Acids Res.

[96] Keshava Prasad TS, Goel R, Kandasamy K, Keerthikumar S, Kumar S, Mathivanan S, Telikicherla D, Raju R, Shafreen B, Venugopal A, Balakrishnan L, Marimuthu A, Banerjee S, Somanathan DS, Sebastian A, Rani S, Ray S, Harrys Kishore CJ, Kanth S, Ahmed M, Kashyap MK, Mohmood R, Ramachandra YL, Krishna V, Rahiman BA, Mohan S, Ranganathan P, Ramabadran S, Chaerkady R, Pandey A (2009). Human Protein Reference Database--2009 update. Nucleic Acids Res.

[97] Huttlin EL, Bruckner RJ, Navarrete-Perea J, Cannon JR, Baltier K, Gebreab F, Gygi MP, Thornock A, Zarraga G, Tam S, Szpyt J, Gassaway BM, Panov A, Parzen H, Fu S, Golbazi A, Maenpaa E, Stricker K, Guha Thakurta S, Zhang T, Rad R, Pan J, Nusinow DP, Paulo JA, Schweppe DK, Vaites LP, Harper JW, Gygi SP (2021). Dual proteome-scale networks reveal cell-specific remodeling of the human interactome. Cell..

[98] Giurgiu M, Reinhard J, Brauner B, Dunger-Kaltenbach I, Fobo G, Frishman G, Montrone C, Ruepp A (2019). CORUM: the comprehensive resource of mammalian protein complexes-2019. Nucleic Acids Res.

[99] Jassal B, Matthews L, Viteri G, Gong C, Lorente P, Fabregat A, Sidiropoulos K, Cook J, Gillespie M, Haw R, Loney F, May B, Milacic M, Rothfels K, Sevilla C, Shamovsky V, Shorser S, Varusai T, Weiser J, Wu G, Stein L, Hermjakob H, D'Eustachio P (2020). The reactome pathway knowledgebase. Nucleic Acids Res.

[100] Huang H-Y, Lin Y-C-D, Li J, Huang K-Y, Shrestha S, Hong H-C (2020). miRTarBase 2020: updates to the experimentally validated microRNA–target interaction database. Nucleic Acids Res.

[101] Xiao F, Zuo Z, Cai G, Kang S, Gao X, Li T (2009). miRecords: an integrated resource for microRNA–target interactions. Nucleic Acids Res.

[102] Csardi G, Nepusz T (2006). The igraph software package for complex network research. InterJ Complex Syst.

[103] Auton A, Abecasis GR, Altshuler DM, Durbin RM, Abecasis GR, Bentley DR (2015). A global reference for human genetic variation. Nature..

[104] Hart T, Moffat J (2016). BAGEL: a computational framework for identifying essential genes from pooled library screens. BMC Bioinformatics.

[105] Ghandi M, Huang FW, Jane-Valbuena J, Kryukov GV, Lo CC, McDonald ER (2019). Next-generation characterization of the Cancer Cell Line Encyclopedia. Nature..

[106] Kanehisa M, Furumichi M, Tanabe M, Sato Y, Morishima K (2016). KEGG: new perspectives on genomes, pathways, diseases and drugs. Nucleic Acids Res.

[107] Wishart DS, Feunang YD, Guo AC, Lo EJ, Marcu A, Grant JR, Sajed T, Johnson D, Li C, Sayeeda Z, Assempour N, Iynkkaran I, Liu Y, Maciejewski A, Gale N, Wilson A, Chin L, Cummings R, le D, Pon A, Knox C, Wilson M (2018). DrugBank 5.0: a major update to the DrugBank database for 2018. Nucleic Acids Res.

[108] Iorio F, Knijnenburg TA, Vis DJ, Bignell GR, Menden MP, Schubert M, Aben N, Gonçalves E, Barthorpe S, Lightfoot H, Cokelaer T, Greninger P, van Dyk E, Chang H, de Silva H, Heyn H, Deng X, Egan RK, Liu Q, Mironenko T, Mitropoulos X, Richardson L, Wang J, Zhang T, Moran S, Sayols S, Soleimani M, Tamborero D, Lopez-Bigas N, Ross-Macdonald P, Esteller M, Gray NS, Haber DA, Stratton MR, Benes CH, Wessels LFA, Saez-Rodriguez J, McDermott U, Garnett MJ (2016). A Landscape of pharmacogenomic interactions in cancer. Cell..

[109] Wagner AH, Walsh B, Mayfield G, Tamborero D, Sonkin D, Krysiak K (2020). A harmonized meta-knowledgebase of clinical interpretations of somatic genomic variants in cancer. Nat Genet.

[110] MySQL 8.0 reference manual. https://dev.mysql.com/doc/refman/8.0/en/.

[111] Bakken S, Suraski Z, Schmid E (2020). PHP manual.

[112] Chang W, Cheng J, Allaire J, Sievert C, Schloerke B, Xie Y (2021). shiny: web application framework for R.

