# Supplementary material for: Comparative assessment of genes driving cancer and somatic evolution in non-cancer tissues: an update of the Network of Cancer Genes (NCG) resource
Source: Genome Biol. 2022 Jan 26;23:35. doi: 10.1186/s13059-022-02607-z (PMC8790917; doi:10.1186/s13059-022-02607-z)
Supplement: Supplementary file 2 — Additional file 2: Figure S1. Literature search, review and annotation workflow; Figure S2. Correlation between numbers of donors and cancer drivers in individual organ systems; Figure S3. Patterns of driver damaging alterations in TCGA samples. [file 13059_2022_2607_MOESM2_ESM.pdf]

Fig. S1. Literature search, review and annotation workflow.

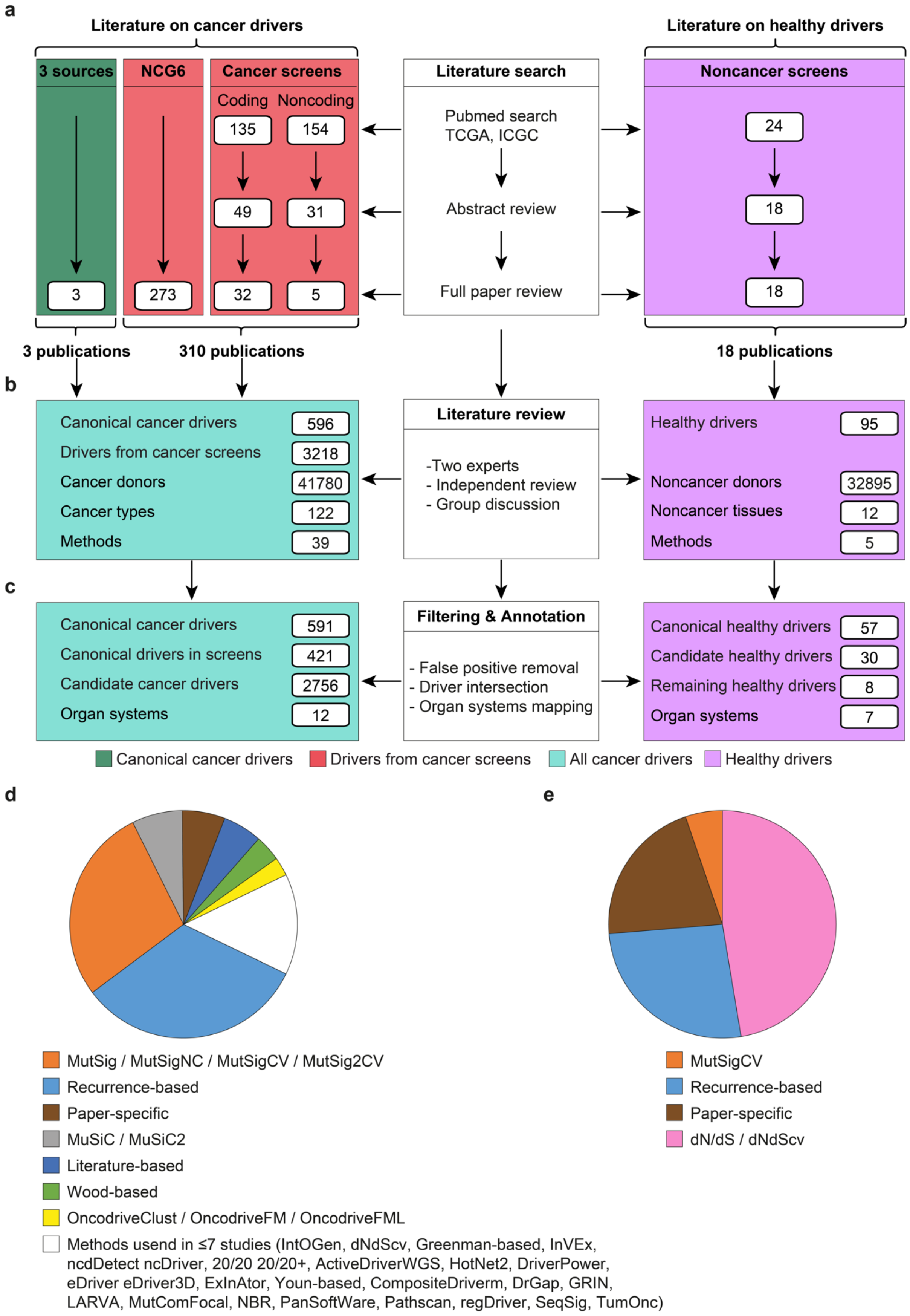

a. Publications reporting cancer and noncancer sequencing screens from the initial literature search in Pubmed, TCGA and ICGC were reviewed at the abstract and full text levels and added to three sources of canonical cancer genes and previously curated papers in NCG 6.0 [1] for a total of 331 publications.

b. Two experts reviewed each publication independently and conflicting annotations were further discussed. Lists of canonical cancer drivers, drivers from cancer and noncancer screens, cancer types and noncancer tissues, and methods used to detect drivers were annotated. Additionally, the number of cancer and noncancer donors were extracted.

c. The resulting lists of drivers were filtered out for possible false positives (**Additional File 3, Table S2**) and intersected to annotate the canonical drivers in cancer screens, candidate cancer drivers (remaining drivers in cancer screens), canonical healthy drivers, candidate healthy drivers, and remaining healthy drivers. Cancer types and noncancer tissues were mapped to organ systems [2]. The full workflow is explained in the Methods.

Usage of driver detection methods across (**d**) cancer and (**e**) noncancer screens. If a screen used several methods, it was counted multiple times. Multiple versions of the same method as well as methods used in less than 8 studies were aggregated. The full list is available in **Additional File 1, Table S1**.

Fig. S2. Correlation between numbers of donors and cancer drivers in individual organ systems

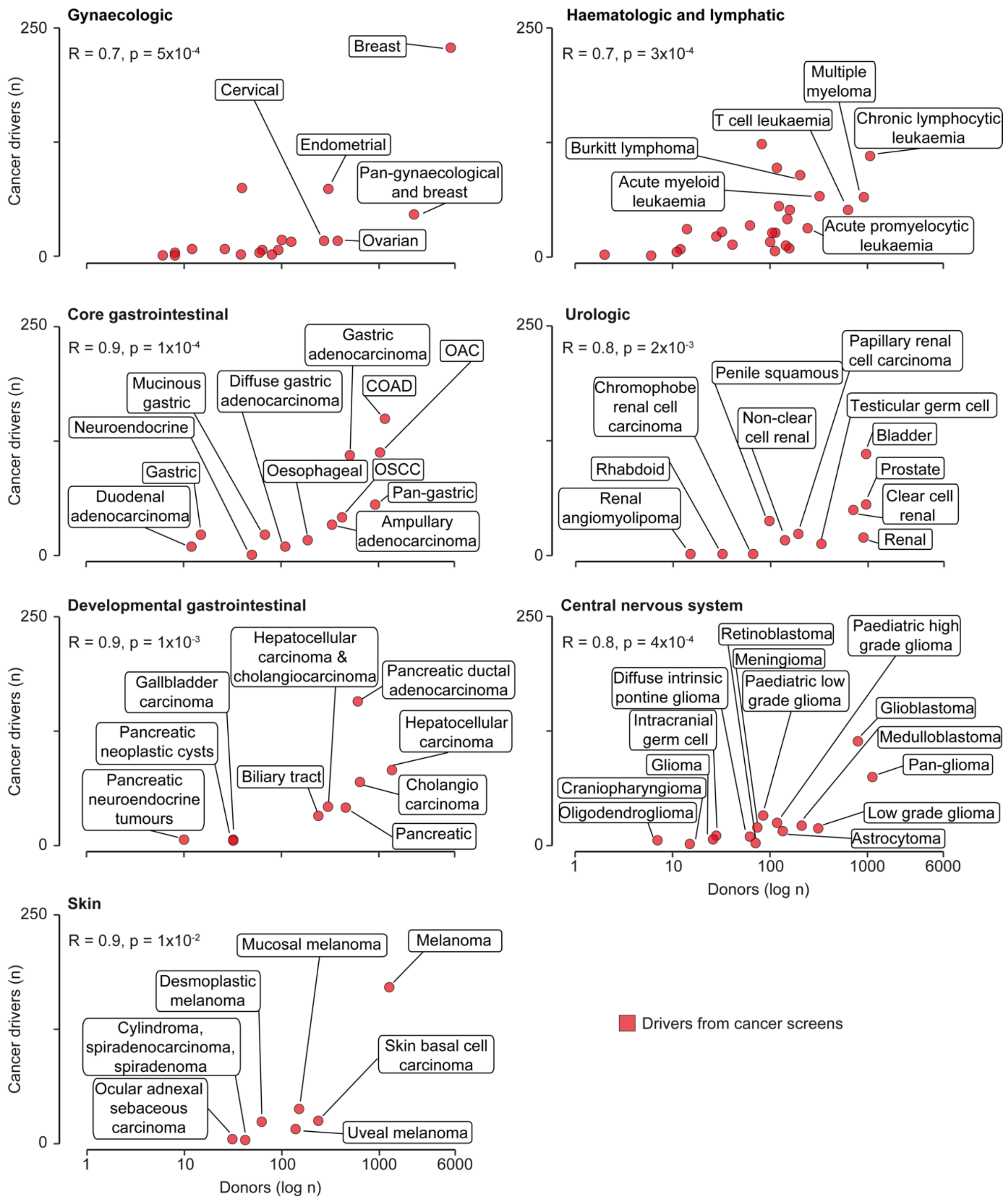

Correlations between numbers of sequenced donors and identified cancer drivers in individual cancer types mapping to each organ system. Only organ systems with significant correlations are reported. Spearman correlation coefficient R and

associated p-value are shown. OAC: oesophageal adenocarcinoma. OSCC: oesophageal squamous cell carcinoma. COAD: colorectal adenocarcinoma.

Fig. S3: Patterns of driver damaging alterations in TCGA samples.

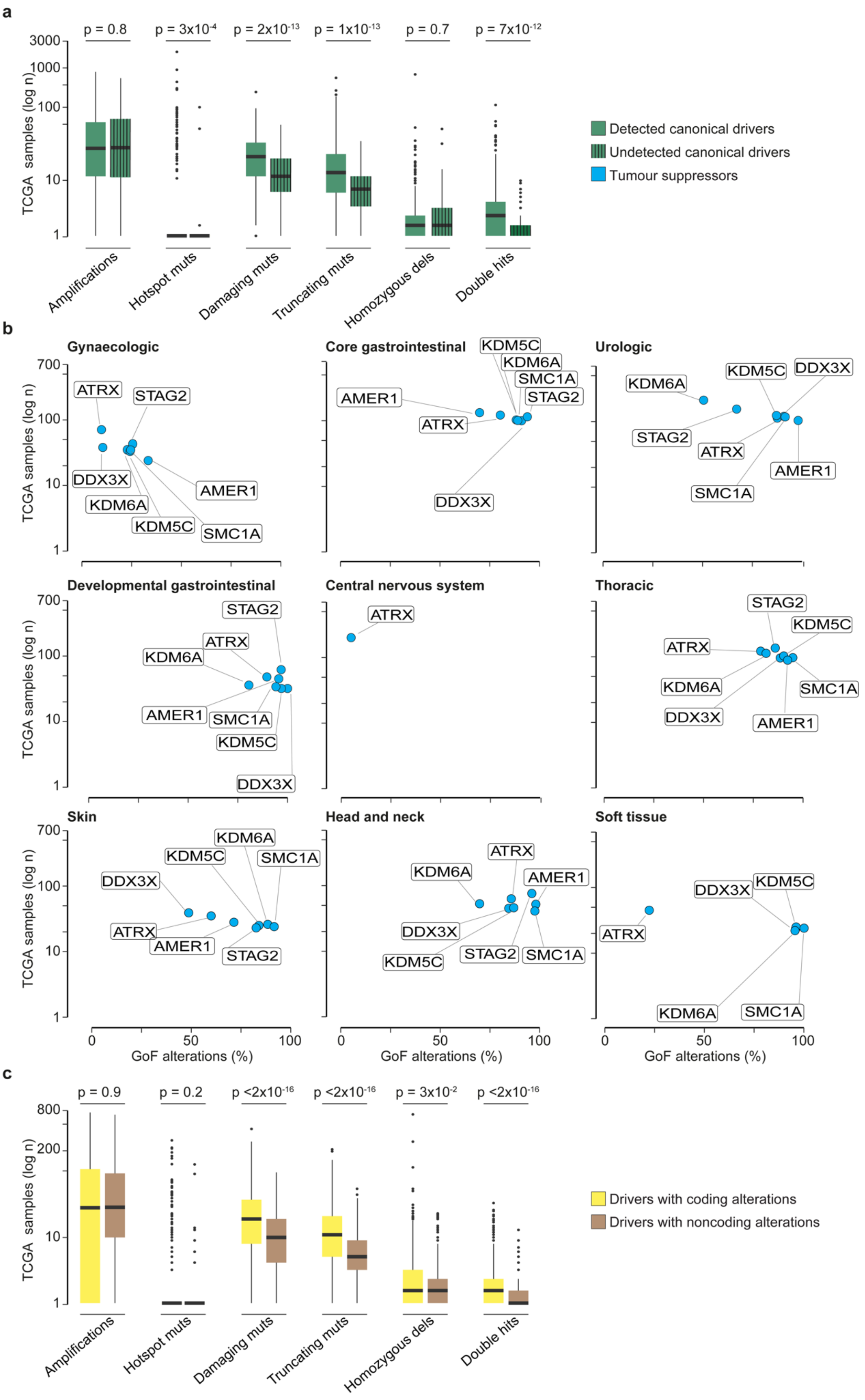

a. Number of TCGA samples with damaging alterations (all, LoF, GoF) in canonical drivers that were detected (421) or undetected (170) by cancer detection methods, divided by type of damaging alterations.

b. Proportion of gain of function (GoF) alterations affecting seven frequently damaged (>500 samples) canonical tumour suppressors. All these genes had an organ system-specific prevalence of GoF and loss of function (LoF) alterations.

c. Number of TCGA samples with damaging alterations in genes previously reported to have coding or only noncoding driver alterations, divided by type of damaging alterations.

All distributions were compared using a two-sided Wilcoxon rank-sum test.

References

1. Repana D, Nulsen J, Dressler L, Bortolomeazzi M, Venkata SK, Tourna A, Yakovleva A, Palmieri T, Ciccarelli FD: **The Network of Cancer Genes (NCG): a comprehensive catalogue of known and candidate cancer genes from cancer sequencing screens.** *Genome Biology* 2019, **20:**1.
2. Hoadley KA, Yau C, Hinoue T, Wolf DM, Lazar AJ, Drill E, Shen R, Taylor AM, Cherniack AD, Thorsson V, et al: **Cell-of-Origin Patterns Dominate the Molecular Classification of 10,000 Tumors from 33 Types of Cancer.** *Cell* 2018, **173:**291-304.e296.
