# Supplementary material for: Comparative assessment of genes driving cancer and somatic evolution in non-cancer tissues: an update of the Network of Cancer Genes (NCG) resource
Source: Genome Biol. 2022 Jan 26;23:35. doi: 10.1186/s13059-022-02607-z (PMC8790917; doi:10.1186/s13059-022-02607-z)
Supplement: Supplementary file 10 — Additional file 10. Review history. [file 13059_2022_2607_MOESM10_ESM.docx]

Review History

**First round of review**

**Reviewer 1**

**Are you able to assess all statistics in the manuscript, including the appropriateness of statistical tests used?**

Yes, and I have assessed the statistics in my report.

**Comments to author:**

I want to congratulate the authors for this massive project and great resources that they have put together. I've been playing with the website and it is very nicely implemented. I think it will become an useful tool for cancer community.

Some minor comments:

1. Have enough healthy screenings been included to identify healthy drivers?

2. The high overlap between the healthy drivers and canonical drivers suggest that multiple triggers will need to be present to initialize oncogenesis. Would that be possible to look at bi-drivers or even tri-drivers occurrence?

3. The inadequate saturation of non-coding variants seems like a confirmation bias toward coding regions. Can authors discuss about how to mitigate this problem?

**Reviewer 2**

**Are you able to assess all statistics in the manuscript, including the appropriateness of statistical tests used?**

There are no statistics in the manuscript.

**Comments to author:**

In this paper Dressler et al report the analysis of cancer drivers and drivers of non-malignant clones in healthy tissue. This work is very timely and provides some important insights. For example, the central role of cancer and healthy drivers in the cell is very insightful among others. I only have a couple of concerns:

1. Major: This concern is related to biases in studies upon which this analysis relies. While the authors perform curation for coding cancer drivers, it seems the conclusions for non-coding drivers are based on partial list of studies published and/or questionable studies. For example, the methods used in Cornish et al and Hayward et al may be less stringent than the ones used in Campbell et al. The authors could perhaps incorporate evidence of experimental validation and/or consensus between studies for non-coding drivers in their analysis and discussion. Some resources like https://cncdatabase.med.cornell.edu/ may be helpful.

2. Minor: The sentence 'Although that this may be affected by the lower number of studies, it may also indicate that the healthy driver repertoire is easier to saturate since less drivers are needed to initiate and sustain noncancer clonal expansion' is not clear since even though the number of drivers needed may be fewer for healthy tissue, they can be unique and the number of unique drivers that may cause growth in healthy tissue might still be large.

**Authors Response**

**Point-by-point responses to the reviewers’ comments:**

Reviewer n.1

I want to congratulate the authors for this massive project and great resources that they have put together. I've been playing with the website and it is very nicely implemented. I think it will become an useful tool for cancer community.

*We thank the reviewer for their kind words and are glad that they find NCG a useful resource for the community.*

Some minor comments:

1. Have enough healthy screenings been included to identify healthy drivers?

*Our analysis of healthy drivers covered all primary sites that had been sequenced at the time of the first submission. At present, the sequencing screens of noncancer tissues are still relatively few. However, the field is moving rapidly, and we will integrate new studies in the future NCG updates. We now mention this in the Discussion (p.26).*

2. The high overlap between the healthy drivers and canonical drivers suggest that multiple triggers will need to be present to initialize oncogenesis. Would that be possible to look at bi-drivers or even tri-drivers occurrence?

*We agree with the reviewer that multiple driver events are likely needed for transformation of somatically mutated clones into cancer.*

*This has been proposed in the literature [1, 2] and is supported by Figure 3G, where almost 60% and 39% of TCGA samples have at least two or three damaged canonical drivers, respectively. If we then consider all drivers (canonical and candidates), the percentages of TCGA samples with at least two or three drivers raise to 66% and 50%, respectively. Therefore, the majority of cancer samples indeed acquire alterations in more than one driver.*

*To address the point of the reviewer more specifically, we calculated the percentages of TCGA samples with damaging alterations in two or more drivers, where at least one was a healthy driver. This resulted in 53% and 37% of TCGA samples, when only canonical drivers were considered and 57% and 46% when all drivers (canonical and candidates) were considered. This is now mentioned in the Results (p.13-14).*

3. The inadequate saturation of non-coding variants seems like a confirmation bias toward coding regions. Can authors discuss about how to mitigate this problem?

*We agree with the reviewer that there is a bias towards protein-coding cancer drivers. As commented in the text (p.5), this is likely due to the overwhelming prevalence of coding sequencing screens (Figure 1A).*

*As we now discuss in the in the text (p.8-9 and 25), this bias can be mitigated by performing additional whole genome sequencing screens and developing methods for the prediction of noncoding drivers.*

Reviewer n.2

In this paper Dressler et al report the analysis of cancer drivers and drivers of non-malignant clones in healthy tissue. This work is very timely and provides some important insights. For example, the central role of cancer and healthy drivers in the cell is very insightful among others. I only have a couple of concerns

*We thank the reviewer for their favourable comments.*

1. Major: This concern is related to biases in studies upon which this analysis relies. While the authors perform curation for coding cancer drivers, it seems the conclusions for non-coding drivers are based on partial list of studies published and/or questionable studies. For example, the methods used in Cornish et al and Hayward et al may be less stringent than the ones used in Campbell et al. The authors could perhaps incorporate evidence of experimental validation and/or consensus between studies for non-coding drivers in their analysis and discussion. Some resources like https://eur03.safelinks.protection.outlook.com/?url=https%3A%2F%2Fcncdatabase.med.cornell.edu%2F&data=04%7C01%7Cfrancesca.ciccarelli%40kcl.ac.uk%7Ce20fd830e10a4ad9dffb08d9bc9da63d%7C8370cf1416f34c16b83c724071654356%7C0%7C0%7C637748208418527474%7CUnknown%7CTWFpbGZsb3d8eyJWIjoiMC4wLjAwMDAiLCJQIjoiV2luMzIiLCJBTiI6Ik1haWwiLCJXVCI6Mn0%3D%7C3000&sdata=54RzHxnfVoDCfPZ8393tO6yJq2ReEugxqpFghFA89N4%3D&reserved=0 may be helpful

*For both coding and noncoding cancer drivers, we performed a comparably rigorous literature curation that, starting from all cancer sequencing screens indexed in ICGC, TCGA and PubMed, led to a set of studies that described coding or noncoding driver alterations hitting protein-coding genes. The criteria for this curation are detailed in the Methods (p.27) and Supplementary Figure 1A.*

*In the case on noncoding mutational screens, we started from 154 papers which were reduced to 19, after manual review. In this respect, we do not think that the list of noncoding studies has been less curated than the one of coding studies. However, we do agree that the knowledge of noncoding driver alterations is still limited and may be prone to false positives. To account for this, we embraced the suggestion of the reviewer to incorporate further support of the driver role of 531 genes hit by noncoding driver alterations only (Figure 1E). We found that only 64 of them have some additional support, while the remaining 467 genes were predicted as drivers only in one screen.*

*This is now commented in the Results (p.5) and in Figure 1F.*

2. Minor: The sentence 'Although that this may be affected by the lower number of studies, it may also indicate that the healthy driver repertoire is easier to saturate since less drivers are needed to initiate and sustain noncancer clonal expansion' is not clear since even though the number of drivers needed may be fewer for healthy tissue, they can be unique and the number of unique drivers that may cause growth in healthy tissue might still be large.

*We have changed the text accordingly (p.9).*

*REFERENCES*

*1. Wijewardhane N, Dressler L, Ciccarelli FD: Normal Somatic Mutations in Cancer Transformation. Cancer Cell 2021, 39:125-129.*

*2. Kakiuchi N, Ogawa S: Clonal expansion in non-cancer tissues. Nat Rev Cancer 2021, 21:239-256.*

**Second round of review**

**Reviewer 1**

The revision is satisfactory.
